# Catalytic Reversible (De)hydrogenation To Rotate a Chemically Fueled Molecular Switch

**DOI:** 10.1002/anie.202214763

**Published:** 2022-11-15

**Authors:** Enzo Olivieri, Na Shao, Roselyne Rosas, Jean‐Valère Naubron, Adrien Quintard

**Affiliations:** ^1^ Aix Marseille Univ CNRS Centrale Marseille iSm2 Marseille France; ^2^ Aix Marseille Univ CNRS Centrale Marseille FSCM Marseille France; ^3^ Univ. Grenoble Alpes CNRS DCM 38000 Grenoble France

**Keywords:** Catalysis, Conformation, Green Chemistry, Hydrogenation, Molecular Switch

## Abstract

We report here the development of a rotating molecular switch based on metal‐catalyzed reversible (de)‐hydrogenation. Under an argon atmosphere, acceptorless dehydrogenation induces a switch from an alcohol to a ketone, while reversing to a hydrogen pressure switches back the system to the alcohol. Based on a tolane scaffold, such reversible (de)‐hydrogenation enables 180° rotation. The absence of waste accumulation in a switch relying on chemical stimuli is of great significance and could potentially be applied to the design of efficient complex molecular machines.

## Graphical abstract

Most chemically fueled molecular switches suffer from the problem of waste accumulation inhibiting the system. Herein, we present a 180° rotating molecular switch based on reversible (de)hydrogenation avoiding waste accumulation. Catalyzed by an iridium complex, switching from a pressure of argon to a pressure of hydrogen triggers a reversible change in the system from alcohol to ketone, promoting the rotation around an alkyne axis.

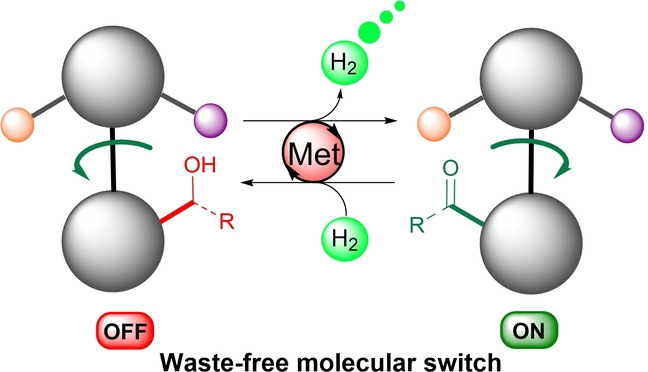

Nature's biomachinery, able to achieve molecular motion in response to a stimulus or chemical fuel, is essential to processes in living organisms. Given the power of these dynamic biological systems, the development of new synthetic molecular machines might provide new opportunities for applications ranging from physics to biology.^[1]^ But despite the intensive research in this area, a long journey is still ahead before these synthetic systems can efficiently compete with nature and be able to selectively promote useful processes. This is why chemists must continuously propose innovative approaches to trigger efficient molecular switches. Among the potential external stimuli, aside from light, heat, or electrical input, a broad array of chemical fuels have been applied with success.^[2]^ However, as a major drawback, most of these chemical stimuli produce undesired chemical waste whose accumulation in the system can rapidly inhibit the machinery.[^2^, ^3^] To overcome this limitation, new approaches excluding waste are needed. Recently, emergent strategies were developed to try to solve this challenge, such as dialysis, or the use of self‐evacuated or inert waste.^[3]^ However, none of these strategies avoid waste formation and the development of new chemical stimuli is mandatory.

Two‐directions redox processes have recently been applied to molecular switches through sequential hydrogenation and oxidation using various chemical reagents.^[4]^ These systems take advantage of aromatization/dearomatization processes through the use of heterogeneous catalysts. Most of these systems are interesting colored switches, but cannot be transposed to the engineering of complex molecular machines. In addition, applying chemical oxidants has the disadvantage of generating waste accumulating in the system that can be detrimental to the good operability of the switch even when oxygen is used which might generate interfering water.

In organic synthesis, waste‐free change in the oxidation state can easily be triggered through alcohols acceptorless dehydrogenation and reversible ketones hydrogenation (Figure 1a).^[5]^

**Figure 1:**
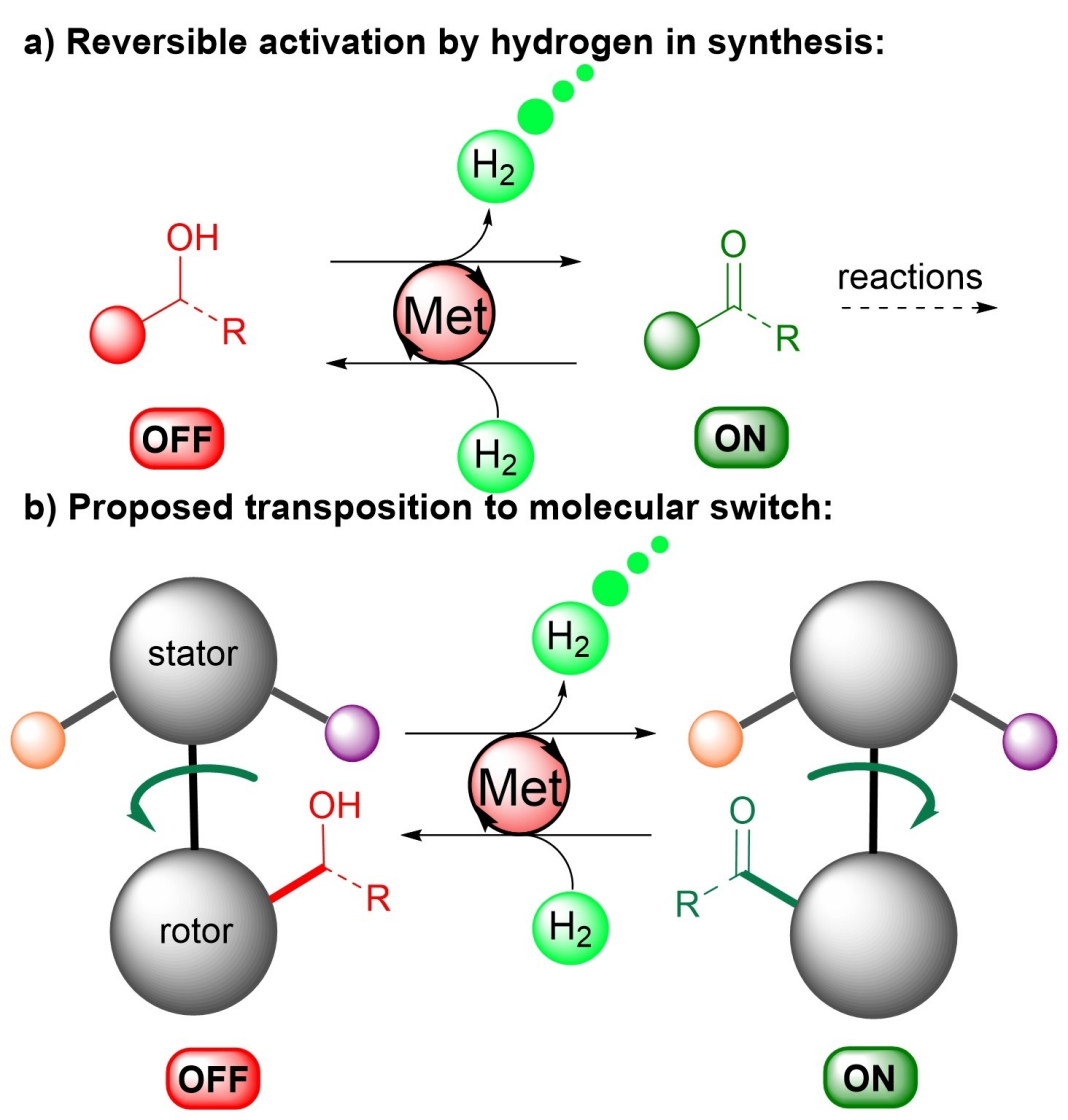
Hydrogen as reversible stimuli for molecular switches.

Promoted by well‐defined metal complexes, these redox reversible processes avoid undesired waste accumulation when switching an organic molecule between two distinct oxidation states. This can be applied to switch unreactive alcohols to reactive carbonyl compounds comparable to an ON/OFF activation pathway.^[5]^ These properties are also central in the field of energy through the storage of hydrogen in various organic molecules such as methanol.[^6^, ^7^] Consequently, it would be highly desirable to transpose such principles to the field of molecular machines.

In this context, we hypothesized that moving reversibly from an alcohol to a ketone through catalytic acceptorless dehydrogenation and hydrogenation could represent a promising platform to build new rotary switches (Figure 1b).

This could be induced by an easy change in the conditions from a hydrogen pressure promoting system reduction, to an argon atmosphere ejecting hydrogen gas from the machine thus avoiding any potential waste accumulation and enabling to displace the system out of equilibrium.^[8]^ As a result, by embedding the redox function on the southern hemisphere of the tolane switch (rotor), a change in the oxidation state would promote a rapid 180° rotation of the rotor through different interactions with the northern hemisphere (stator).

In order to develop a first model of molecular switch, we choose to take advantage of a rapid rotation around an alkyne in a tolane scaffold, which had been used with success notably by the group of Hamilton in different 180° conformational switches.^[9]^ For such purpose, finding an appropriate alcohol function able to switch easily and reversibly to the ketone represented an important challenge and would be facilitated using secondary alcohols.^[8]^ Consequently, we designed tolane molecular switches, possessing on one hemisphere a secondary benzylic alcohol. The other hemisphere would be constituted of an amide able to coordinate to the ketone. At first, we focused on the synthesis and application of alkyne **1_ketone_** possessing only one substituent on the northern hemisphere. However, we discovered that such alkyne lacked of stability during the hydrogenation process. In addition, single crystal X‐Ray diffraction revealed that, minimizing steric interactions, both ketone and amide functions were located on the undesired opposite sides (Figure 2).^[10]^

**Figure 2:**
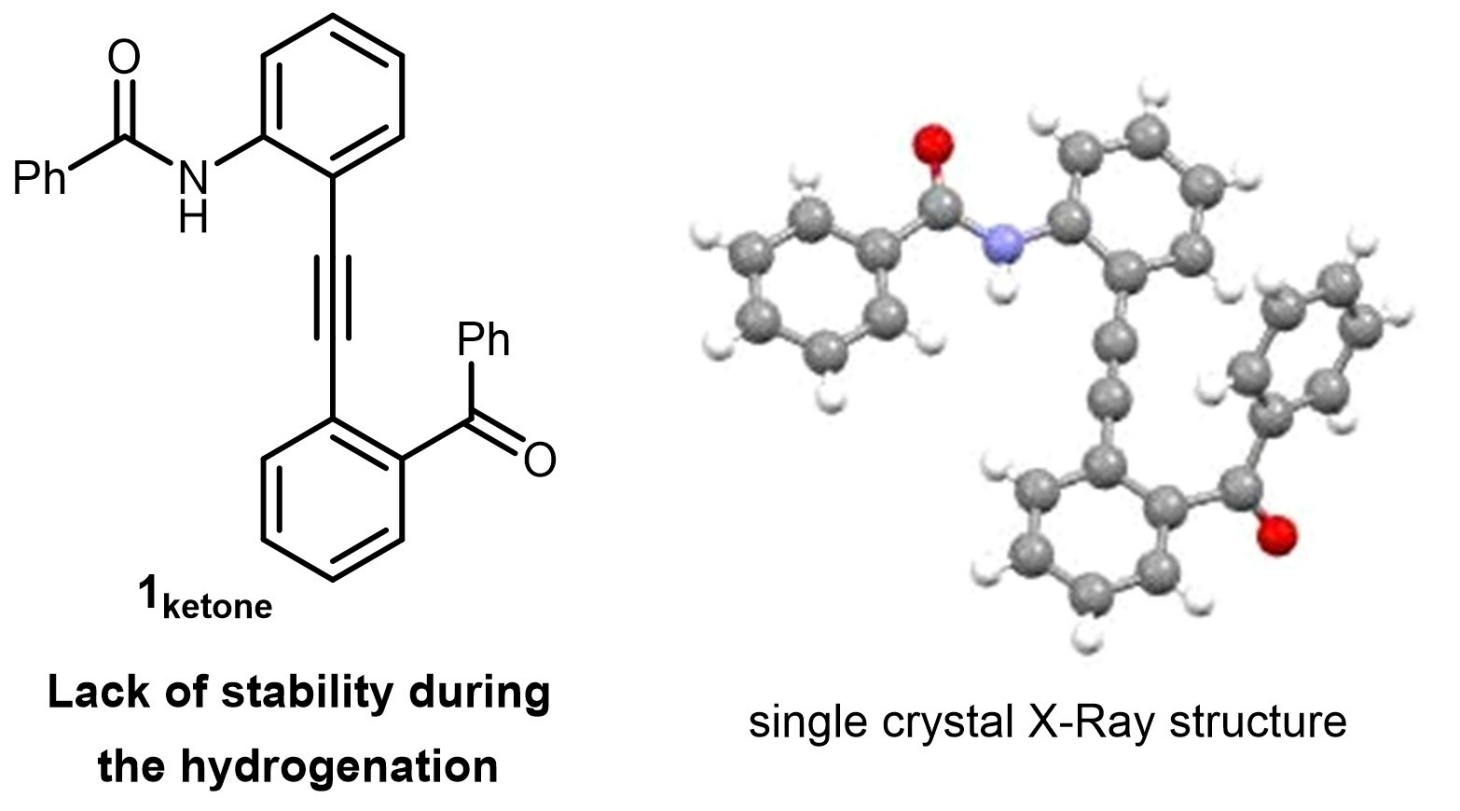
First molecular switch model prepared.

In order to stabilize the structure and bring the conformation of the ketone in interaction with the amide, we designed another model switch bearing an additional electron‐rich dimethylamino group on the northern hemisphere (Figure 3a). In addition, insertion of a dimethylamino group could also stabilize the desired conformation at the alcohol level. The desired molecular switch **2_ketone_** was prepared efficiently in a 7 longest linear steps sequence converging through a final Suzuki coupling (see Figure S1).

**Figure 3:**
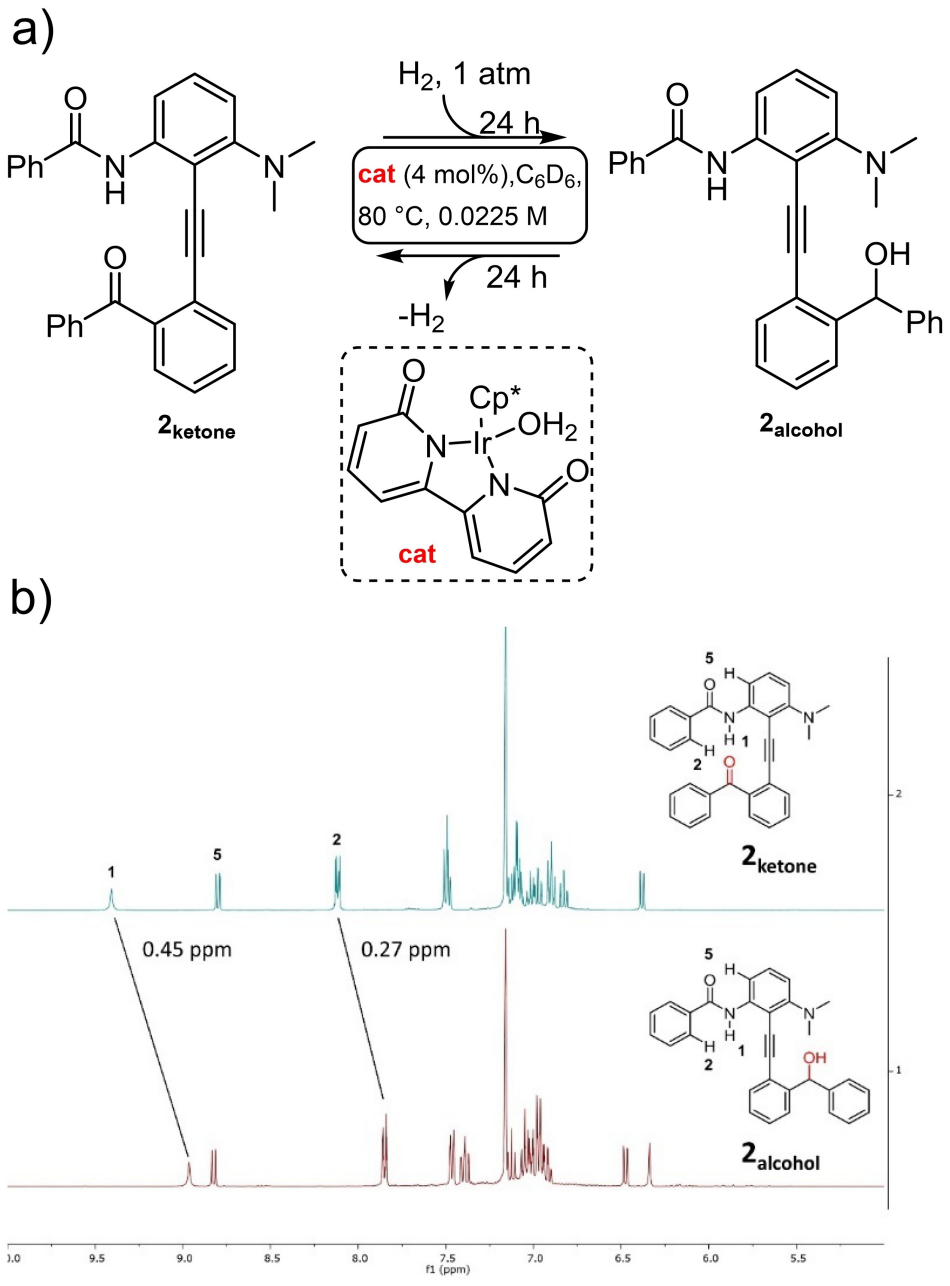
a) Operation of the switch **2** through hydrogenation/acceptorless dehydrogenation. b) ^1^H NMR spectra of **2_ketone_** and **2_alcohol_** in C_6_D_6_ (0.045 M).

In order to promote the desired switch, we used the iridium catalyst **cat** developed by the group of Yamaguchi, notably able to trigger acceptorless dehydrogenation under mild conditions.^[8d]^ Deuterated benzene is used as solvent since it is compatible with both steps and not entering in competition with the potential interactions between each hemispheres. From **2_ketone_**, applying 1 atmosphere of hydrogen, a clean conversion to **2_alcohol_** was observed after 24 hours and the structure in the solid state determined by single crystal X‐Ray diffraction (Figure 4).^[10]^ Simply changing the hydrogen atmosphere with an argon one, a clean return to **2_ketone_** is observed after 24 hours under the otherwise identical conditions. Of interest, only 4 mol % of the iridium catalyst is enough to promote both reactions despite the use of the relatively diluted conditions (0.0225 M) applied in order to avoid potential intermolecular interactions.

**Figure 4:**
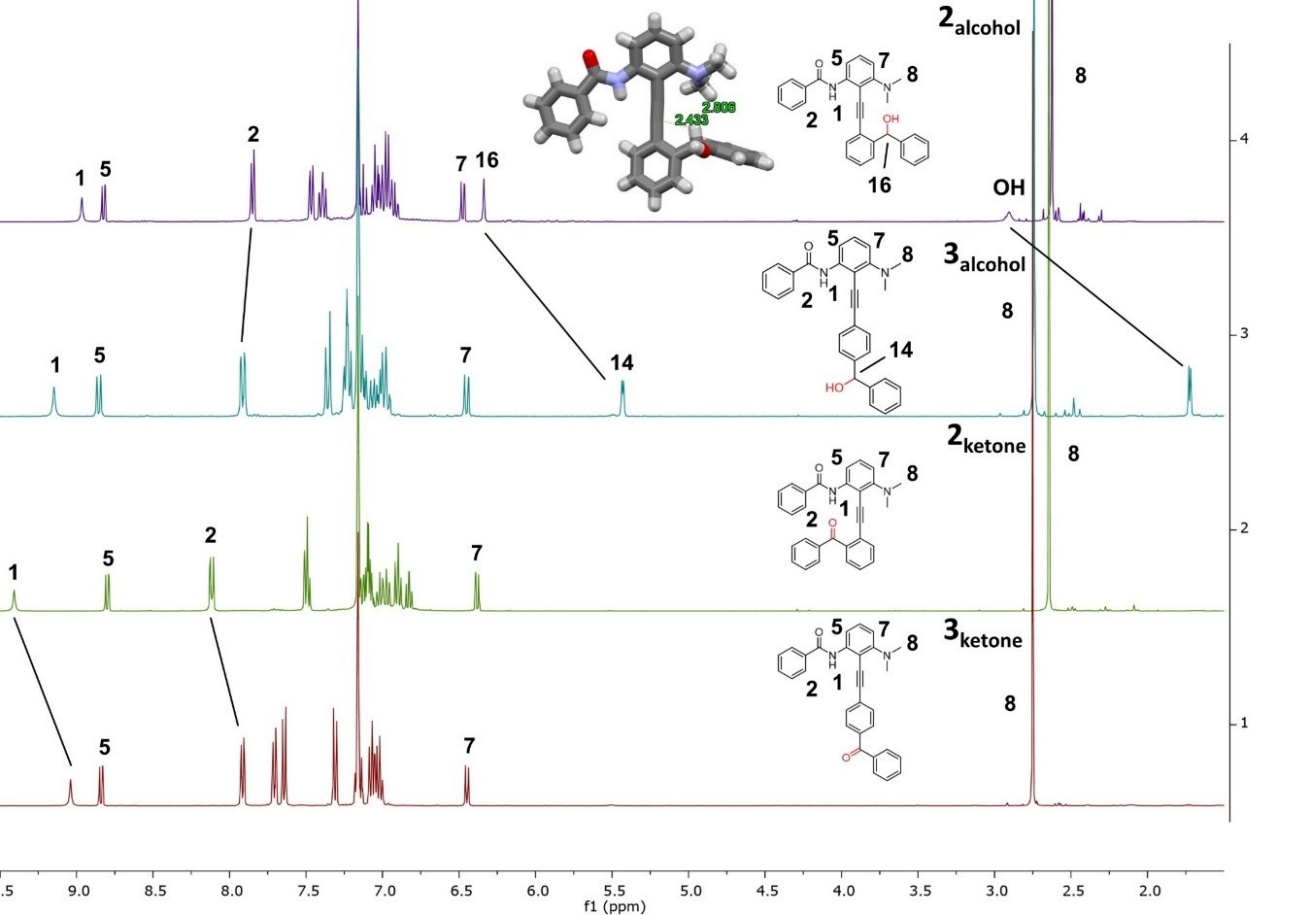
^1^H NMR spectra of **2_alcohol_**, **3_alcohol_**, **2_ketone_**, **3_ketone_** and single‐crystal X‐ray structure of **2_alcohol._**

^1^H NMR enables to follow easily the system through the two states (Figure 3b). Most notably, an important change in the chemical shift of protons **1** (−0.45 ppm) and **2** (−0.27 ppm) can be observed when switching from **2_ketone_** *to* **2_alcohol_**. Amide chemical shifts had been extensively used by the group of Hamilton to characterize tolanes switches.^[9]^ Since these protons are not affected by a change in the electronic character of the substituent (vide infra), this is in agreement with an important structural change in the conformation during the switch. In addition, the absence of change in the chemical shift of proton **5**, confirm that the amide is locked and that this upfield proton (8.7 ppm) is interacting with the carbonyl of the amide in a rigid 6‐member ring. Consequently, this indicates that on **2_ketone_**, the ketone is predominantly interacting with the amide through hydrogen bonding, while on **2_alcohol_**, a 180° rotation brings the alcohol on the opposite side, far from the amide.

These observations were also confirmed by variable temperature NMR analysis (Figure S9, S10). In order to further determine the exact conformation of **2_ketone_** and **2_alcohol_**, a complete conformational analysis was performed corroborating NMR and IR spectroscopy supported by DFT calculations.

First of all, the impact of the 180° rotation around the alkyne over the chemical shift was confirmed through the preparation of para‐substituted hemisphere references **3_ketone_** and **3_alcohol_**. These two compounds are close in electronic character to **2_ketone_** and **2_alcohol_**_,_ but cannot create any hydrogen bond between the lower and upper parts of the switch. This enables to confirm the potential interactions observed in **2_ketone_** and **2_alcohol_** (Figure 4). Most notably, at the alcohol oxidation state, a 0.93 ppm shift is observed on the benzylic protons **14** and **16** between **2_alcohol_** and **3_alcohol_**. In **2_alcohol_** the observation of this proton at 6.34 ppm, already observed on other tolanes,^[10]^ is due to a strong anisotropic effect. This effect is corroborated on the calculated DFT major conformations (Figure S16, conformation A1 and A2) where the proton **16** is coplanar with the adjacent aromatic ring and the minor ones where proton **16** is colinear to the alkyne axis (Figure S16, conformation A3 and A4). NOESY experiments on **2_alcohol_**, gave interactions between the benzylic proton and the NMe_2_ (See Figure S4), once again confirming the 180° nature of the switch. At the ketone oxidation state, chemical shift of the amide protons **1** and **2** considerably changed between **2_ketone_** and **3_ketone_**. Indeed, the hydrogen bonding interaction between the amide and the ketone lead to an increase of 0.37 ppm in the chemical shift of proton **1** between **2_ketone_** and **3_ketone_**. This result is comparable with previous literature increase in chemical shifts observed through such hydrogen bonding (Figure S11).

At last, comparison of the theoretical IR spectra of **2_alcohol_** and **2_ketone_** with the calculated one from the Boltzman distribution of the different conformations found in DFT (Figure S14–S22), confirmed the nature of the rotating switch and the major conformers populations (Figure S14–S22). These results confirm that on **2_ketone_**, stabilization occurs through hydrogen bonding between the ketone and the amide together with a repulsion between the electron‐rich amine and the ketone. **2_alcohol_** conformations result from multiple small stabilizations (H⋅⋅⋅NMe_2_, hydrogen bonding, π‐stacking, van der Waals). The sum of these small interactions favor exclusively the conformers with the alcohol on the side of the dimethylamine with a >99 : 1 calculated Boltzmann distribution.

Overall, the system can be described as a catalyzed switch evolving between two states. Given the rate of both hydrogenation and dehydrogenation reactions (around 24 hours to go to completion), much higher than the fast rotation around the alkyne (<1 s, Figure S23), the kinetic of the system exclusively depends on the iridium catalyzed transformation. Considering the thermodynamic, the ketone represents the most favored state and can be formed in 100 % through the use of a large vessel enabling sufficient dilution of the generated H_2_. The thermodynamic displacement from the ketone to the alcohol is favored through the use of an excess of hydrogen, displacing the system out of equilibrium and enabling the exclusive formation of **2_alcohol_**.

Finally, we checked the possibility to run consecutive (de)‐hydrogenation switch cycles. Gratifyingly, starting from **2_ketone_** and by using only 4 mol % of iridium **cat**, two consecutive acceptorless dehydrogenation/hydrogenation cycles could be performed (see Figure S2). It is important to notice that these iridium catalyzed processes are performed in one‐pot, without any other implication of the operator aside from the change in the gas atmosphere. After these two cycles, the third dehydrogenation takes longer (6 days) to go to almost completion (86 % conv), indicating the partial deactivation of the iridium catalyst with the additional appearance of small amount of an unknown impurity. The catalyst deactivation was confirmed when repeating the cycles and adding additional catalyst when a decrease in dehydrogenation rate is observed. This second catalyst addition restored the catalytic activity providing again full conversion (Figure S3). This indicates the actual limits of the system that should be improved through the potential application of catalysts of improved reactivity and the design of less sensitive switches.

To conclude, we presented herein our success at developing a 180° rotational switch based on catalyzed hydrogenation/acceptorless dehydrogenation. As a result of the reversible catalytic approach used, this switch using a chemical stimuli does not generate any accumulating waste. This represents the first step towards the elaboration of complex molecular machines based on such general principle. In order to improve the reliability of the system over multiple cycles, future directions should focus on the modification of the switch scaffold limiting its degradation and on the implementation of metal catalysts of improved efficiency. In addition, the kinetic and thermodynamic of the switch of the system might also be modulated through the change in the alcohol substitution.

## Conflict of interest

The authors declare no conflict of interest.

## Supporting information

As a service to our authors and readers, this journal provides supporting information supplied by the authors. Such materials are peer reviewed and may be re‐organized for online delivery, but are not copy‐edited or typeset. Technical support issues arising from supporting information (other than missing files) should be addressed to the authors.

Supporting Information

Supporting Information

Supporting Information

Supporting Information

## Data Availability

The data that support the findings of this study are available in the Supporting Information of this article.
