# Supplementary material for: Catalytic Reversible (De)hydrogenation To Rotate a Chemically Fueled Molecular Switch
Source: Angew Chem Int Ed Engl. 2022 Nov 15;61(50):e202214763. doi: 10.1002/anie.202214763 (PMC10099969; doi:10.1002/anie.202214763)
Supplement: Supplementary file 1 — Supporting Information [file ANIE-61-0-s002.pdf]

Angewandte Chemie
Eine Zeitschrift der Gesellschaft Deutscher Chemiker

Supporting Information

Catalytic Reversible (De)hydrogenation To Rotate a Chemically Fueled Molecular Switch

*E. Olivieri, N. Shao, R. Rosas, J.-V. Naubron, A. Quintard**

Supporting Information

Table of contents

**General Information**	**2**
**Tolanes Synthesis**	**3**
**Molecular switch experiments**	**12**
**NMR analysis**	**15**
**Single crystal X-Ray analysis**	**23**
**IR analysis and Theoretical calculations**	**24**
**NMR spectra**	**35**
**References**	**43**

General information

Reactions were run under argon atmosphere or if specified inside a glovebox (Jacomex GP (concept)-II-P) in oven-dried glassware.

Analytical thin layer chromatography (TLC) was performed on silica gel 60 F254 aluminum plates (Macherey-Nagel) containing a 254 nm fluorescent indicator. TLC plates were visualized by exposure to short wave ultraviolet light (254 nm) and further visualization was achieved by staining $KMnO_4$ and heating by a hot air gun. Flash column chromatography was performed using silica gel (35–70 µm, 60 Å, Acros). Organic extracts were dried over anhydrous Na_2SO_4.

Microwave experiments were carried out with an Antor Paar Microwave Synthesis Reactor, Monowave 300 apparatus and calibrations were performed every four months

NMR spectra were recorded on a Bruker AC 300 (300 MHz), a Bruker AC 400 (400 MHz) and a Bruker 500 (500 MHz) spectrometer. Chemical shifts are given in ppm, using as internal standards the residual $CHCl_3$ or $C_6D_5H_1$ signal for ^1H NMR (δ= 7.26, δ= 7.16) and the deuterated solvent signal for ^{13}C NMR (δ= 77.16, δ= 128.06). Data for ^{13}C NMR are reported as follows: chemical shift (multiplicity). Data for ^1H NMR are reported as follows: chemical shift (multiplicity [s = singlet, d = doublet, t = triplet, tt = triplet of triplet, q = quadruplet, m = multiplet, brs = broad singlet], coupling constants *J* in Hertz (Hz), integration). Anhydrous solvent (THF, CH_2Cl_2, toluene, acetonitrile) was obtained from a Solvent Purification System M Braun SPS-800.

High resolution mass spectra (HRMS) were performed on a QstarElite (Applied Biosystems SCIEX) spectrometer equipped with atmospheric pression ionization source (API) pneumatically assisted. Samples were ionized by positive electrospray mode as follows: electrospray tension (ISV): 5500 V; opening tension (OR): 50 V; nebulization gas pression (air): 20 psi. IR spectra were recorded using a Thermo Nicolet Avatar 330 FT-IR instrument (Thermo Nicolet Corporation, Madison, WI, USA).

Iron >99%, reduced, powder (fine) was purchased from Merck. Iridium catalyst (cat) was synthetized according to the Yamaguchi procedure.[1] Unless specified, commercial reagents were used as received.

Tolanes synthesis

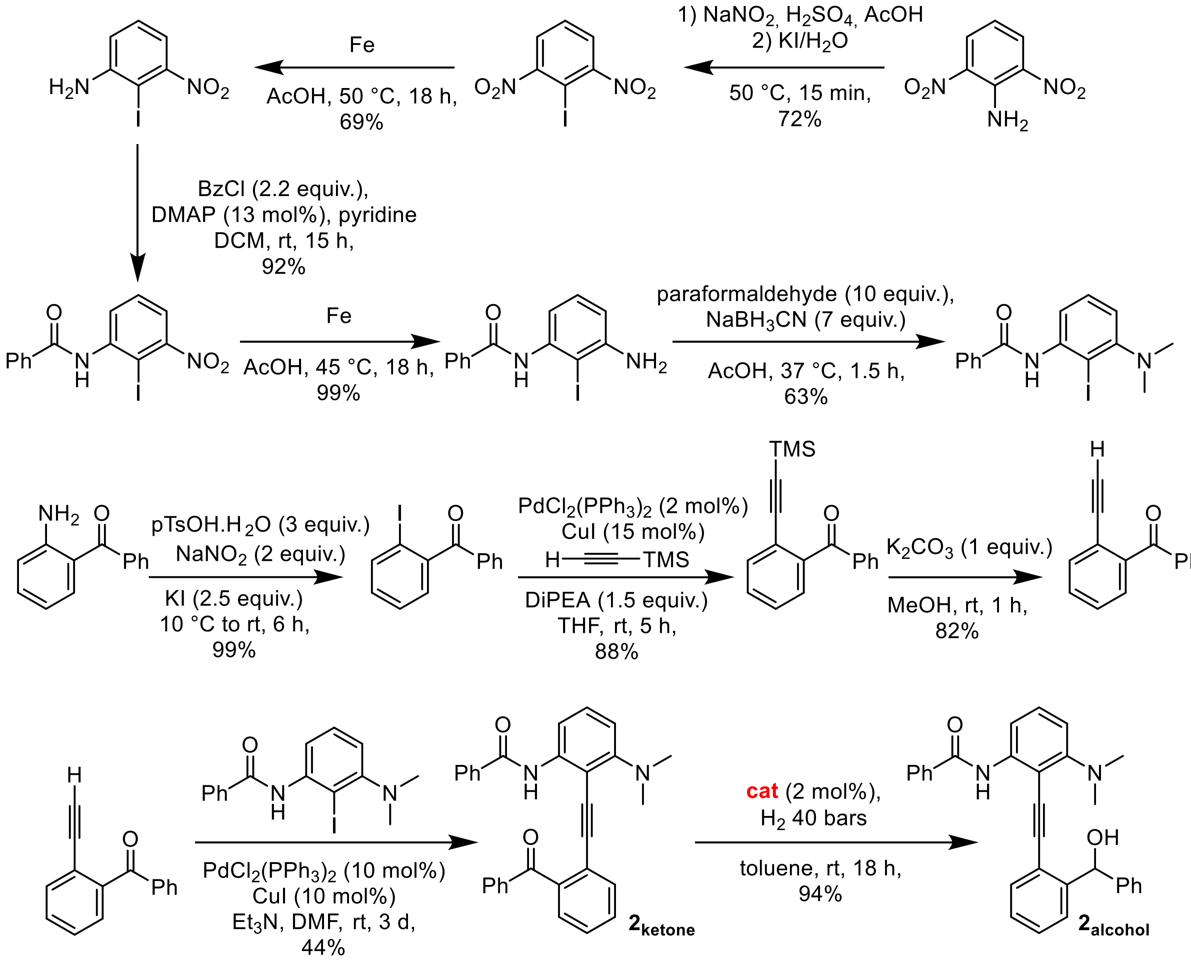

***Figure S1**: Synthesis route for **2**$_{ketone}$ and **2**$_{ketone}$. For **3**$_{ketone}$ and **3**$_{ketone}$, (4-ethynylphenyl)(phenyl)methanone was used for the final Sonogashira coupling.*

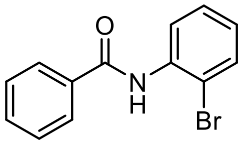

N-(2-bromophenyl)benzamide:

The product was synthesized according to a published procedure.[2]

^{1}H NMR (400 MHz, CDCl$_3$) δ(ppm): 8.56 (dd, *J* = 8.3, 1.6 Hz, 1H), 8.47 (s, 1H), 7.97 – 7.92 (m, 2H), 7.62 – 7.50 (m, 4H), 7.42 – 7.34 (m, 1H), 7.02 (ddd, *J* = 8.0, 7.4, 1.6 Hz, 1H). Data are in accordance with the literature.[2]

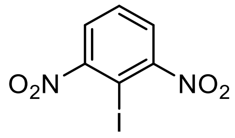

2,6-Dinitroiodobenzene:

According to a modified procedure,[3] an oven-dried 250 mL 3-neck round bottom flask equipped with a thermometer was charged with sulfuric acid (9 mL) at 30 °C. Sodium nitrite was added portionwise and the reaction mixture temperature kept below 45 °C and stirred for 0.5 h. Then a solution of 2,6-Dinitroaniline (2.00 g, 11.0 mmol, 1.00 equiv) in AcOH (40 mL), was added dropwise to the reaction mixture and the reaction mixture temperature kept below 40 °C. After 0.5 h a solution of KI (2.1 g, 12.65 mmol, 1.15 equiv) in water (25 mL) at 50 °C was added to the reaction mixture, the temperature kept below 50 °C. After 15 min the reaction mixture was poured into water (200 mL) at 0 °C, the resulting precipitate was filtered and washed three times with water. The resulting solid was dissolved in CH_2Cl_2 (200 mL) and the resulting solution was washed with sodium thiosulfate (2 x 100 mL), dried over Na_2SO_4, filtered and the solvent evaporated. The product was isolated as an orange solid (2.34 g, 7.96 mmol, 72% yield).

TLC (cyclohexane/EtOAc 4:1) R*f* 0.33

^{1}H NMR (400 MHz, $CDCl_3$) δ(ppm): 7.83 (d, *J* = 8.0 Hz, 2H), 7.66 (t, *J* = 8.0 Hz, 1H). Data are in accordance with the literature.[3]

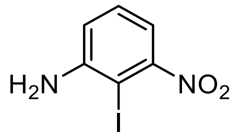

2-iodo-3-nitroaniline:

According to a modified procedure,[4] 2,6-Dinitroiodobenzene (2.3 g, 7.84 mmol, 1.00 equiv) was dissolved in AcOH (23 mL) under argon and stirred at 50 °C. Then Fe powder (1.26 g, 22.56 mmol, 2.88 equiv) was added at once, and the solution was stirred for 18 h, added to a saturated solution of $NaHCO_3$ (400 mL), and extracted with ethyl acetate (3 x 250 mL). The combined organic layers were dried over Na_2SO_4, filtered and the solvent evaporated, the crude product was purified over silica gel (gradient ethyl acetate/cyclohexane (0/100 to 30/70). The product was isolated as an orange solid (1.42 g, 5.38 mmol, 69% yield).

TLC (cyclohexane/EtOAc 4:1) R*f* 0.28

^{1}H NMR (400 MHz, $CDCl_3$) δ(ppm): 7.21 (t, *J* = 8.0 Hz, 1H), 7.05 (dd, *J* = 7.9, 1.4 Hz, 1H), 6.88 (dd, *J* = 8.1, 1.4 Hz, 1H), 4.54 (brs, 2H). Data are in accordance with the literature.[4]

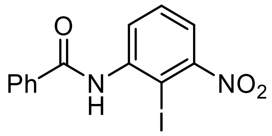

N-(2-iodo-3-nitrophenyl)benzamide:

According to a modified procedure,[4] 2-iodo-3-nitroaniline (0.80 g, 3.03 mmol, 1.00 equiv), DMAP (49 mg, 0.40 mmol, 0.13 equiv), pyridine (0.26 mL, 3.22 mmol, 1.06 equiv) were dissolved in CH_2Cl_2 (3 mL) under argon atmosphere. Then benzoyl chloride (0.78 mL, 6.71 mmol, 2.21 equiv) was added and the solution was stirred for 18 h, before being quenched with 2 M HCl (40 mL), extracted with CH_2Cl_2 (3 x 60 mL). The combined organic layers were washed with a saturated solution of $NaHCO_3$ (3 x 60 mL), dried over Na_2SO_4, filtered and the solvent evaporated, the crude product was purified by recrystallisation in EtOAc. The product was isolated as a yellow solid (1.03 g, 2.72 mmol, 92% yield).
TLC (cyclohexane/EtOAc 17:3) R*f* 0.55
^{1}H NMR (400 MHz, $CDCl_3$) δ(ppm) 8.74 – 8.71 (m, 1H), 8.64 (brs, 1H), 8.02 – 7.98 (m, 2H), 7.67 – 7.61 (m, 1H), 7.60 – 7.51 (m, 5H). Data are in accordance with the literature.[4]

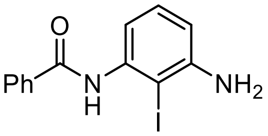

N-(3-amino-2-iodophenyl)benzamide:

N-(2-iodo-3-nitrophenyl)benzamide (660 mg, 1.79 mmol, 1.00 equiv) was dissolved in AcOH (7.7 mL) under argon and stirred at 45 °C. Then Fe powder (330 mg, 5.91 mmol, 3.30 equiv) was added at once, and the solution was stirred for 18 h, added to a saturated solution of $NaHCO_3$ (200 mL), and extracted with EtOAc (3 x 100 mL). The combined organic layers were dried over Na_2SO_4, filtered and the solvent evaporated, the crude product was used without further purification. The product was isolated as a brown solid (605 mg, 1.79 mmol 99% yield).
TLC (cyclohexane/EtOAc 17:3) R*f* 0.22
^{1}H NMR (400 MHz, $CDCl_3$) δ(ppm) 8.27 (brs, 1H), 8.03 – 7.92 (m, 2H), 7.76 (dd, *J* = 8.0, 1.4 Hz, 1H), 7.61 – 7.45 (m, 3H), 7.15 (t, *J* = 8.0 Hz, 1H), 6.57 (dd, *J* = 8.0, 1.4 Hz, 1H), 4.35 – 4.05 (brs, 2H). Data are in accordance with the literature.[4]

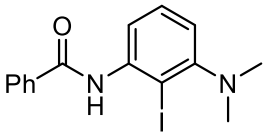

N-(3-(dimethylamino)-2-iodophenyl)benzamide:

N-(3-amino-2-iodophenyl)benzamide (605 mg, 1.79 mmol, 1.00 equiv) was dissolved in AcOH (12.5 mL) under argon and stirred at 37 °C. Then paraformaldehyde (538 mg, 17.9 mmol, 10.0 equiv) was added at once, and after 10 min, $NaBH_3CN$ (475 mg, 12.6 mmol, 7.00 equiv) was added portionwise. The solution was allowed to stir for 8 h, and was then poured into a saturated solution of $NaHCO_3$ (600 mL) and extracted with DCM (3 x 150 mL). The combined organic layers were dried over Na_2SO_4 filtered and the solvent evaporated. The crude product was purified over silica gel gradient ethyl acetate/cyclohexane (0/100 to 30/70). The product was isolated as an orange solid (414 mg, 1.13 mmol, 63% yield).
TLC (cyclohexane/EtOAc 4:1) R*f* 0.59

m.p. 95.8 °C

^{1}H NMR (400 MHz, C_6D_6) δ(ppm): 8.77 (dd, *J* = 8.2, 1.4 Hz, 1H), 8.58 (brs, 1H), 8.01 – 7.94 (m, 2H), 7.13 – 7.02 (m, 4H), 6.53 (dd, *J* = 7.9, 1.5 Hz, 1H), 2.42 (s, 6H).

^{13}C NMR (101 MHz, $CDCl_3$) δ (ppm): 164.8 (C(O)NH), 155.9 ©, 140.6 ©, 135.5 ©, 131.8 (CH), 129.8 (CH), 128.9 (2CH), 127.5 (2CH), 117.6 ©, 116.9 (CH), 95.6 ©, 45.0 (2CH_3).

HRMS (ESI) calculated for $[C_{15}H_{15}IN_2O+H]^+$: 367.0302, found: 367.0294.

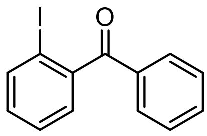

(2-iodophenyl)(phenyl)methanone:

According to a modified procedure,[5] an oven-dried 250 mL 3-neck round bottom flask equipped with a thermometer was charged with (2-aminophenyl)(phenyl)methanone (2.00 g, 10.1 mmol, 1.00 equiv) and *p*-TsOH.H_2O (5.90 g, 30.42 mmol, 3.00 equiv) in acetonitrile (30 mL). The solution was stirred 5 min at 10 °C, then $NaNO_2$ (1.40 g, 20.3 mmol, 2 equiv) was added followed by KI (4.21 g, 25.35 mmol, 2.50 equiv), the solution was allowed to stir for 6 h. The resulting red solution was quenched with $Na_2SO_2O_3$ until the reaction mixture turned yellow, extracted with DCM (3 x 150 mL). The combined organic layers were dried over Na_2SO_4, filtered and the solvent evaporated. The crude product was purified over silica gel (gradient ethyl acetate/cyclohexane (0/100 to 30/70). The product was isolated as an orange oil (3.17 g, 10.1 mmol, 99% yield).

TLC (cyclohexane/EtOAc 3:2) R*f* 0.89

^{1}H NMR (400 MHz, $CDCl_3$) δ(ppm) 7.93 (dd, *J* = 7.9, 1.1 Hz, 1H), 7.83 – 7.78 (m, 2H), 7.60 (tt, *J* = 7.6 Hz, 1.2 Hz, 1H), 7.46 (m, 3H), 7.30 (dd, *J* = 7.6, 1.7 Hz, 1H), 7.18 (td, *J* = 7.7, 1.7 Hz, 1H). Data are in accordance with the literature.[6]

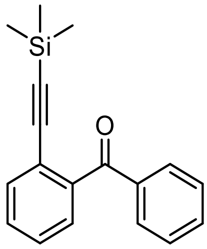

Phenyl(2-((trimethylsilyl)ethynyl)phenyl)methanone:

According to a modified procedure,[7] an oven-dried 20 mL pressure vial was charged with (2-iodophenyl)(phenyl)methanone (616 mg, 2.00 mmol, 1.00 equiv), $Pd(Cl)_2(PPh_3)_2$ (28.0 mg, 0.04 mmol, 0.02 equiv), CuI (8.00 mg, 0.04 mmol, 0.02 equiv), and PPh_3 (6.00 mg, 0.02 mmol, 0.01 equiv) under argon atmosphere. Then a degassed solution of THF (1 mL) and freshly distilled triethylamine (10 mL) were added, and the reaction mixture was stirred for 2 min. (trimethylsiyl)acetylene was added at once and the reaction mixture was allowed to stir for 18 h. Then the solvent was evaporated and the resulting mixture dissolved in CH_2Cl_2 (100 mL), filtered over a patch of celite, dried over Na_2SO_4, filtered and

evaporated. The crude product was purified over silica gel (gradient ethyl acetate/cyclohexane (0/100 to 20/80). The product was isolated as a brown solid (557 mg, 2.00 mmol, 99% yield).

TLC (cyclohexane/EtOAc 9:1) R*f* 0.59

^{1}H NMR (300 MHz, $CDCl_3$) δ(ppm): 7.84 – 7.78 (m, 2H), 7.61 – 7.51 (m, 2H), 7.49 – 7.40 (m, 5H), -0.06 (s, 9H). Data are in accordance with the literature.[38]

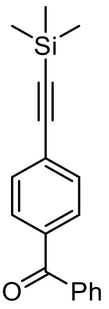

Phenyl(4-((trimethylsilyl)ethynyl)phenyl)methanone:

According to a modified procedure,[7] an oven-dried 20 mL pressure vial was charged with 4-bromobenzophenone (260 mg, 1.00 mmol, 1.00 equiv), $Pd(Cl)_2(PPh_3)_2$ (14.0 mg, 0.02 mmol, 0.02 equiv), CuI (4.00 mg, 0.02 mmol, 0.02 equiv), and PPh_3 (3.00 mg, 0.01 mmol, 0.01 equiv) under argon atmosphere. Then a degassed solution of THF (1 mL) and freshly distilled triethylamine (10 mL) were added, and the reaction mixture was stirred for 2 min. (trimethylsiyl)acetylene was added at once and the reaction mixture was allowed to stir for 18 h at 50 °C. Then the solvent was evaporated and the resulting mixture dissolved in CH_2Cl_2 (100 mL), filtered over a patch of celite, dried over Na_2SO_4, filtered and evaporated. The crude product was purified over silica gel (gradient ethyl acetate/cyclohexane (0/100 to 20/80). The product was isolated as a brown solid (278 mg, 1.00 mmol, 99% yield).

TLC (cyclohexane/EtOAc 4:1) R*f* 0.71

^{1}H NMR (400 MHz, $CDCl_3$) δ(ppm): 7.76 (m, 4H), 7.64 – 7.54 (m, 3H), 7.52 – 7.46 (m, 2H), 0.28 (s, 9H). Data are in accordance with the literature.[7]

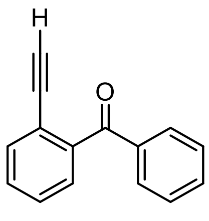

(2-ethynylphenyl)(phenyl)methanone:

According to a modified procedure[4] an 50 mL round bottom flask was charged with phenyl(2-((trimethylsilyl)ethynyl)phenyl)methanone (125 mg, 0.45 mmol, 1.00 equiv) in MeOH (4 mL) under argon atmosphere. Then K_2CO_3 (62.0 mg, 0.45 mmol, 1.00 equiv) was added at once and the reaction mixture was allowed to stir for 90 min. H_2O (10 mL) was added to the reaction mixture, followed by a precipitate formation, then the solid was filtered on a fritted funnel to remove the solution. The precipitate was dissolved in EtOAc (20 mL) dried over Na_2SO_4, filtered and the solvent evaporated and the crude product

was used without further purification. The product was isolated as a dark solid (92.0 mg, 0.45 mmol, 99%).

TLC (cyclohexane/EtOAc 9:1) R*f* 0.53

^{1}H NMR (400 MHz, C_6D_6) δ(ppm): 7.87 – 7.78 (m, 2H), 7.40 – 7.34 (m, 1H), 7.14 – 7.07 (m, 2H), 7.05 – 6.98 (m, 2H), 6.92 – 6.80 (m, 2H), 2.58 (s, 1H). Data are in accordance with the literature.[9]

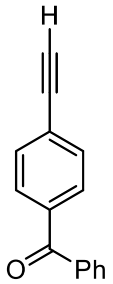

(4-ethynylphenyl)(phenyl)methanone:

According to a modified procedure[4] an 50 mL round bottom flask was charged with Phenyl(4-((trimethylsilyl)ethynyl)phenyl) (278 mg, 1.00 mmol, 1.00 equiv) in MeOH (9 mL) under argon atmosphere. Then K_2CO_3 (138.5 mg, 1.00 mmol, 1.00 equiv) was added at once and the reaction mixture was allowed to stir for 90 min. H_2O (30 mL) was added to the reaction mixture, followed by a precipitate formation, then the solid was filtered on a fritted funnel to remove the solution. The precipitate was dissolved in EtOAc (60 mL) dried over Na_2SO_4, filtered and the solvent evaporated, the crude product was used without further purification. The product was isolated as a dark solid (206 mg, 1.00 mmol, 99%).

TLC (cyclohexane/EtOAc 18:2) R*f* 0.58

^{1}H NMR (400 MHz, C_6D_6) δ(ppm): 7.63 – 7.59 (m, 2H), 7.50 – 7.46 (m, 2H), 7.31 – 7.26 (m, 2H), 7.14 – 7.09 (m, 1H), 7.05 – 6.99 (m, 2H), 2.78 (s, 1H). Data are in accordance with the literature.[10]

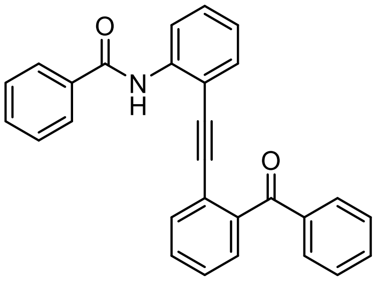

N-(2-((2-benzoylphenyl)ethynyl)phenyl)benzamide (1_{ketone}):

An oven-dried 20 mL MW vial was charged with N-(2-bromophenyl)benzamide (220 mg, 0.800 mmol, 1.00 equiv), (2-ethynylphenyl)(phenyl)methanone (375 mg, 1.82 mmol, 2.27 equiv), $Pd(Cl)_2(PPh_3)_2$ (60.0 mg, 0.0855 mmol, 0.11 equiv), CuI (15 mg, 0.079 mmol, 0.10 equiv). Then the vial was set under inert atmosphere with 3 vacuum-argon cycles. Finally, freshly degassed DMF (6 mL) and triethylamine (6 mL) were added to the vial, and the reaction mixture was heated with a MW to 110 °C for 2 h. The reaction mixture was condensed, dissolved in CH_2Cl_2 and filtered through a pad of celite, and

evaporated. The crude product was purified over silica gel (gradient ethyl acetate/cyclohexane (0/100 to 10/90). The product was isolated as a yellow/orange oil (57 mg, 0.142 mmol, 18% yield).

TLC (cyclohexane/EtOAc 9:1) R*f* 0.30

^{1}H NMR (400 MHz, C_6D_6) δ(ppm): 9.27 (brs, 1H), 9.07 (dd, *J* = 8.5, 1.0 Hz, 1H), 8.15 – 8.09 (m, 2H), 7.54 – 7.49 (m, 2H), 7.42 (ddd, *J* = 7.7, 1.2, 0.4 Hz, 1H), 7.26 (ddd, *J* = 7.7, 1.6, 0.4 Hz, 1H), 7.15 – 7.02 (m, 6H), 6.97 (dd, *J* = 7.7, 1.4 Hz, 1H), 6.95 – 6.91 (m, 2H), 6.83 (td, *J* = 7.6, 1.2 Hz, 1H), 6.70 (td, *J* = 7.6, 1.1 Hz, 1H).

^{13}C NMR (75 MHz, C_6D_6) δ(ppm): 195.25 (*C*(O)), 165.98 (*C*(O)NH), 141.11 (C(O)NH*C*), 140.55 (*C*C(O)), 137.68 (*C*C(O)), 136.05 (*C*C(O)NH), 133.32 (*C*H), 132.92 (*C*H), 132.20 (*C*H), 131.50 (*C*H), 130.74 (*C*H), 130.48 (*C*H), 130.46 (2*C*H), 130.02 (*C*H), 128.57 (2*C*H), 128.47 (2*C*H), 128.35 (2*C*H), 127.92 (*C*H), 123.24 (*C*H), 122.67 (*C*C≡C), 120.45 (*C*H), 112.13 (*C*C≡C), 94.86 (*C*≡C), 90.55 (*C*≡C).

X-Ray: Single X-Ray structure analysis confirmed the structure: CCDC N°2125544.

When performing the hydrogenation on this substrate using the iridium catalyst, rapid decomposition was observed.

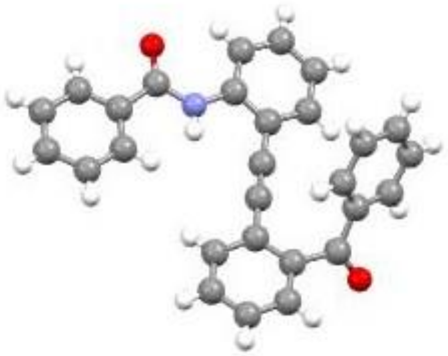

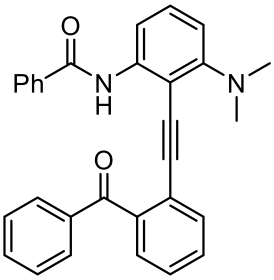

N-(2-((2-benzoylphenyl)ethynyl)-3-(dimethylamino)phenyl)benzamide (2_{ketone}):

According to a modified procedure,[4] a 30 mL Schlenk tube inside a glove box was charged with N-(3-(dimethylamino)-2-iodophenyl)benzamide (366 mg, 1.00 mmol, 1.00 equiv), (2-ethynylphenyl)(phenyl)methanone (412.0 mg, 2.00 mmol, 2.00 equiv), $Pd(Cl)_2(PPh_3)_2$ (70.0 mg, 0.10 mmol, 0.10 equiv) in a degassed solution (freeze-pump-thaw) of DMF (10 mL) and freshly distilled triethylamine (10 mL). The resulting solution was stirred 2 min. Then CuI (19.0 mg, 0.1 mmol, 0.10 equiv) was added and the reaction mixture was allowed to stir until completion. The reaction mixture was condensed, dissolved in CH_2Cl_2 and filtered through a pad of celite, and evaporated. The crude product was purified over silica gel (gradient ethyl acetate/cyclohexane (0/100 to 20/80). The product was

isolated as an orange oil (197 mg, 0.44 mmol, 44% yield). All attempts at crystalizing this compound failed.

TLC (cyclohexane/EtOAc 4:1) R*f* 0.30

^{1}H NMR (400 MHz, C_6D_6) δ(ppm): 9.41 (brs, 1H), 8.80 (dd, *J* = 8.3, 0.8 Hz, 1H), 8.17 – 8.06 (m, 2H), 7.56 – 7.42 (m, 3H), 7.15 – 7.12 (m, 1H), 7.12 – 7.06 (m, 4H), 7.02 (tt, *J* = 6.77, 1.26 Hz, 1H), 6.97 (td, *J* = 7.6, 1.4 Hz, 1H), 6.90 (tt, *J* = 6.7, 1.1 Hz, 2H), 6.83 (td, *J* = 7.6, 1.2 Hz, 1H), 6.38 (dd, *J* = 8.1, 0.9 Hz, 1H) 2.64 (s, 6H).

^{13}C NMR (101 MHz, $CDCl_3$) δ (ppm): 195.2 (*C*(O)), 166.0 (*C*(O)NH), 156.2 (*C*N(CH_3)$_2$), 142.5 (C(O)NH*C*), 140.3 (*C*C(O)), 137.7 (*C*C(O)), 136.4 (*C*C(O)NH), 133.2 (*C*H), 132.9 (*C*H), 131.3 (*C*H), 130.7 (*C*H), 130.6 (*C*H), 130.5 (2*C*H), 129.8 (*C*H), 128.5 (2*C*H), 128.4 (2*C*H), 128.3 (2*C*H), 127.6 (*C*H), 123.2 (*C*C≡C), 113.2 (*C*H), 112.5 (*C*H), 104.7 (*C*C≡C), 100.3 (*C*≡C), 90.2 (*C*≡C), 43.5 (2*C*H$_3$).

HRMS (ESI) calculated for [$C_{30}H_{24}N_2O_2$+H]$^+$: 445.1911, found: 445.1907.

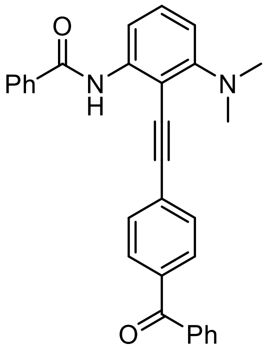

N-(2-((4-benzoylphenyl)ethynyl)-3-(dimethylamino)phenyl)benzamide (3_{ketone}):

According to a modified procedure,[4] a 20 mL Schlenk tube inside a glove box was charged with N-(3-(dimethylamino)-2-iodophenyl)benzamide (210 mg, 0.57 mmol, 1.00 equiv), (4-ethynylphenyl)(phenyl)methanone (206 mg, 1.00 mmol, 1.75 equiv), Pd(Cl)$_2$(PPh$_3$)$_2$ (40.0 mg, 57.0 µmol, 0.10 equiv) in a degassed solution (freeze-pump-thaw) of DMF (5 mL) and freshly distilled triethylamine (5 mL) the resulting solution was stirred 2 min. Then CuI (11.0 mg, 57.0 µmol, 0.10 equiv) was added and the reaction mixture was allowed to stir until completion. The reaction mixture was condensed, dissolved in CH_2Cl_2 and filtered through a pad of celite, and evaporated. The crude product was purified over silica gel (gradient ethyl acetate/cyclohexane (0/100 to 20/80). The product was isolated as an orange oil (217 mg, 0.49 mmol, 86% yield).

TLC (cyclohexane/EtOAc 4:1) R*f* 0.41

^{1}H NMR (400 MHz, C_6D_6) δ(ppm): 9.04 (brs, 1H), 8.84 (dd, *J* = 8.3, 0.6 Hz, 1H), 7.93 – 7.89 (m, 2H), 7.73 – 7.69 (m, 2H), 7.66 – 7.62 (m, 2H), 7.34 – 7.28 (m, 2H), 7.18 – 7.13 (m, 2H), 7.11 – 6.98 (m, 5H), 6.45 (dd, *J* = 8.3, 0.7 Hz, 2H), 2.75 (s, 6H).

^{13}C NMR (101 MHz, C_6D_6) δ(ppm): 194.8 (*C*(O)), 164.8 (*C*(O)NH), 156.1 (*C*N(CH_3)$_2$), 141.8 ©, 138.0 ©, 137.7 ©, 136.0 ©, 132.4 (*C*H), 131.8 (*C*H), 131.1 (*C*H), 131.0 (2*C*H), 130.4 (2*C*H), 130.2 (2*C*H), 128.9 (2*C*H), 128.5 (2*C*H), 127.4 (2*C*H), 127.2 ©, 112.7 (*C*H), 112.3 (*C*H), 104.0 ©, 101.9 ©, 88.3 ©, 43.4 (2*C*H$_3$).

HRMS (ESI) calculated for $[C_{30}H_{24}N_2O_2+H]^+$: 445.1911, found: 445.1906.

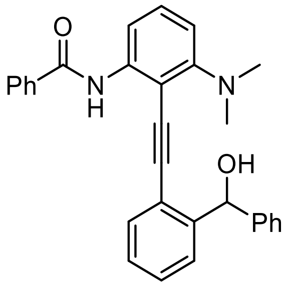

N-(3-(dimethylamino)-2-((2-(hydroxy(phenyl)methyl)phenyl)ethynyl)phenyl)benzamide ($2_{alcohol}$):

To a 5 mL flask was added N-(2-((2-benzoylphenyl)ethynyl)-3-(dimethylamino)phenyl)benzamide (50.0 mg, 112.0 µmol, 1.00 equiv), **cat** (1.30 mg, 2.44 µmol, 0.02 equiv) in toluene (1 mL). The reaction mixture was placed inside an autoclave apparatus with a 40 bars pressure of H_2 and allowed to stir for 18 h. Then the reaction mixture was concentrated and the crude product was purified over silica gel (gradient ethyl acetate/cyclohexane (0/100 to 30/70). The product was isolated as an orange solid (47.0 mg, 105.0 µmol, 94% yield).

TLC (cyclohexane/EtOAc 16:4) R*f* 0.21

m.p. 69.1 °C

^{1}H NMR (400 MHz, C_6D_6) δ(ppm): 8.96 (brs, 1H), 8.82 (dd, *J* = 8.3, 0.8 Hz, 1H), 7.89 – 7.83 (m, 2H), 7.49 – 7.44 (m, 2H), 7.43 – 7.36 (m, 2H), 7.13 (t, *J* = 8.3 Hz, 1H), 7.08 – 6.94 (m, 7H), 6.94 – 6.89 (dd, *J* = 7.5, 1.5 Hz, 1H), 6.48 (dd, *J* = 8.2, 0.9 Hz, 1H), 6.34 (s, 1H), 2.90 (brs, 1H), 2.62 (s, 6H).

^{13}C NMR (101 MHz, C_6D_6) δ(ppm): 165.0 (*C*(O)NH), 156.3 (*C*N(CH_3)$_2$), 145.0 (*C*C(OH)), 143.8 (*C*C(OH)), 141.4 (C(O)NH*C*), 135.8 (*C*C(O)NH), 132.2 (*C*H), 131.7 (*C*H), 130.7 (*C*H), 129.3 (*C*H), 128.9 (2*C*H), 128.5 (2*C*H), 127.8 (*C*H), 127.6 (*C*H), 127.5 (*C*H), 127.5 (2*C*H), 127.1 (2*C*H), 121.9 (*C*C≡C), 113.6 (*C*H), 113.4 (*C*H), 106.1 (*C*C≡C), 100.6 (*C*≡C), 89.5 (*C*≡C), 74.2 (*C*(OH)), 43.9 (2*C*H_3).

HRMS (ESI) calculated for $[C_{30}H_{26}N_2O_2+H]^+$: 447.2067, found: 447.2063.

X-Ray: Single X-Ray structure analysis confirmed the structure: CCDC N° 2125541

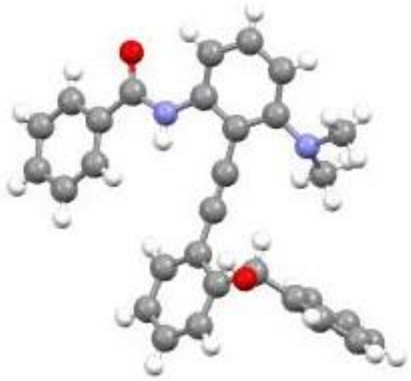

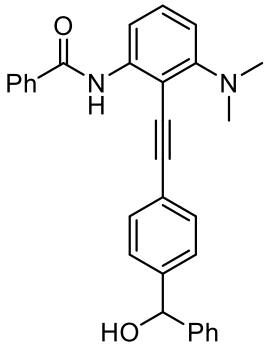

N-(3-(dimethylamino)-2-((4-(hydroxy(phenyl)methyl)phenyl)ethynyl)phenyl)benzamide ($3_{alcohol}$):

To a 5 mL flask was added N-(2-((4-benzoylphenyl)ethynyl)-3-(dimethylamino)phenyl)benzamide (100 mg, 225 µmol, 1.00 equiv), cat (1.30 mg, 2.44 µmol, 0.01 equiv) in toluene (2 mL). The reaction mixture was placed inside an autoclave apparatus with a 40 bars pressure of H_2 and allowed to stir for 18 h. Then the reaction mixture was concentrated and the crude product was purified over silica gel (gradient ethyl acetate/cyclohexane (0/100 to 30/70). The product was isolated as an orange solid (93 mg, 209 µmol, 93% yield).

TLC (cyclohexane/EtOAc 4:1) R*f* 0.22

m.p. 82.5 °C

^{1}H NMR (300 MHz, C_6D_6) δ(ppm): 9.15 (brs, 1H), 8.85 (dd, *J* = 8.3, 0.7 Hz, 1H), 7.91 (dt, *J* = 6.6, 1.5 Hz, 2H), 7.40 – 7.32 (m, 2H), 7.27 – 7.20 (m, 4H), 7.14 – 7.10 (m, 2H), 7.09 – 6.94 (m, 4H), 6.45 (dd, *J* = 8.3, 0.8 Hz, 1H), 5.43 (d, *J* = 3.3 Hz, 1H), 2.74 (s, 6H), 1.72 (d, *J* = 3.5 Hz, 1H).

^{13}C NMR (75 MHz, C_6D_6) δ(ppm): 164.8 (*C*(O)NH), 155.8 (*C*N(CH_3)$_2$), 145.3 ©, 144.4 ©, 141.6 ©, 136.0 ©, 131.7 (*C*H), 131.3 (2*C*H), 130.5 (*C*H), 128.8 (2*C*H), 128.7 (2*C*H), 127.9 (*C*H), 127.4 (2*C*H), 127.1 (2*C*H), 127.0 (2*C*H), 122.4 ©, 112.7 (*C*H), 112.3 (*C*H), 104.6 ©, 102.7 ©, 85.3 ©, 75.9 (*C*(OH)), 43.4 (2*C*H_3).

HRMS (ESI) calculated for $[C_{30}H_{26}N_2O_2+H]^+$: 447.2067, found: 447.2068.

X-Ray: Single X-Ray structure analysis confirmed the structure: CCDC N° 2125543

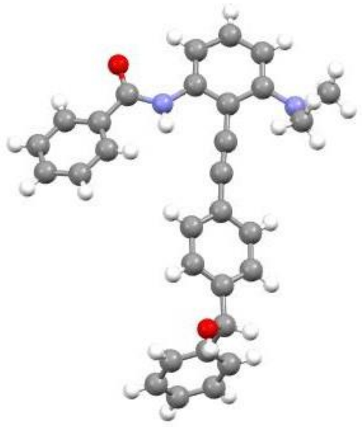

Molecular switch experiments

Optimization of $2_{alcohol}$ acceptorless dehydrogenation:

To a 10 mL Schlenk equipped with a cooler (3 °C) was added N-(3-(dimethylamino)-2-((2-(hydroxy(phenyl)methyl)phenyl)ethynyl)phenyl)benzamide, **cat**, C_6D_6. On top of the cooler was inserted in a septum 3 argon balloons (double jacked, the needle used was an 18 G (1.2 mm diameter)). The reaction mixture was heated to the desired temperature with an oil bath. To follow the conversion the reaction mixture was analyzed by ^1H NMR.

Entry	T (°C)	Solent	Time (h)	cat (mol%)	C (M)	Conversion (%)a
1	37	toluene	24	7	0.038	0
2	50	C_6D_6	40	5	0.045	75
3	80[b]	C_6D_6	24	4	0.0225	>99

Table S1: *Dehydrogenation trial of* $\mathbf{2}_{alcohol}$*.* [a]*, Determined by* ^1H *NMR.* [b]*, Reflux of* C_6D_6 *with oil bath at 115 °C.*

Molecular switch through multiple cycles:

To a 10 mL Schlenk equipped with a cooler (3 °C) was added $\mathbf{2}_{alcohol}$ (60.0 mg, 0.135 mmol, 1.00 equiv), **cat** (3.00 mg, 5.64 µmol, 0.04 equiv) in C_6D_6 (6 mL). On top of the cooler was inserted in a septum 3 gas balloons (double jacked) filled with argon for the oxidation process or hydrogen for the hydrogenation (the needle used was an 18 G (1.2 mm diameter)). The reaction mixture was heated at reflux (80 °C) in an oil bath (115 °C). To follow the conversion, a 0.1 mL sample of the reaction mixture is diluted with 0.4 mL of C_6D_6 and analyzed by ^1H NMR (**Figure S2**). When the desired conversion is obtained, the backward reaction can be setup by removing the 3 balloons, and flushing the reaction mixture for 30 seconds with the other gas. Then, 3 new balloons filled with the desired gas were added. Flushing is mandatory to perform a rapid equilibrium between the gas/solution phase.

NMR monitoring of the molecular switch cycles:

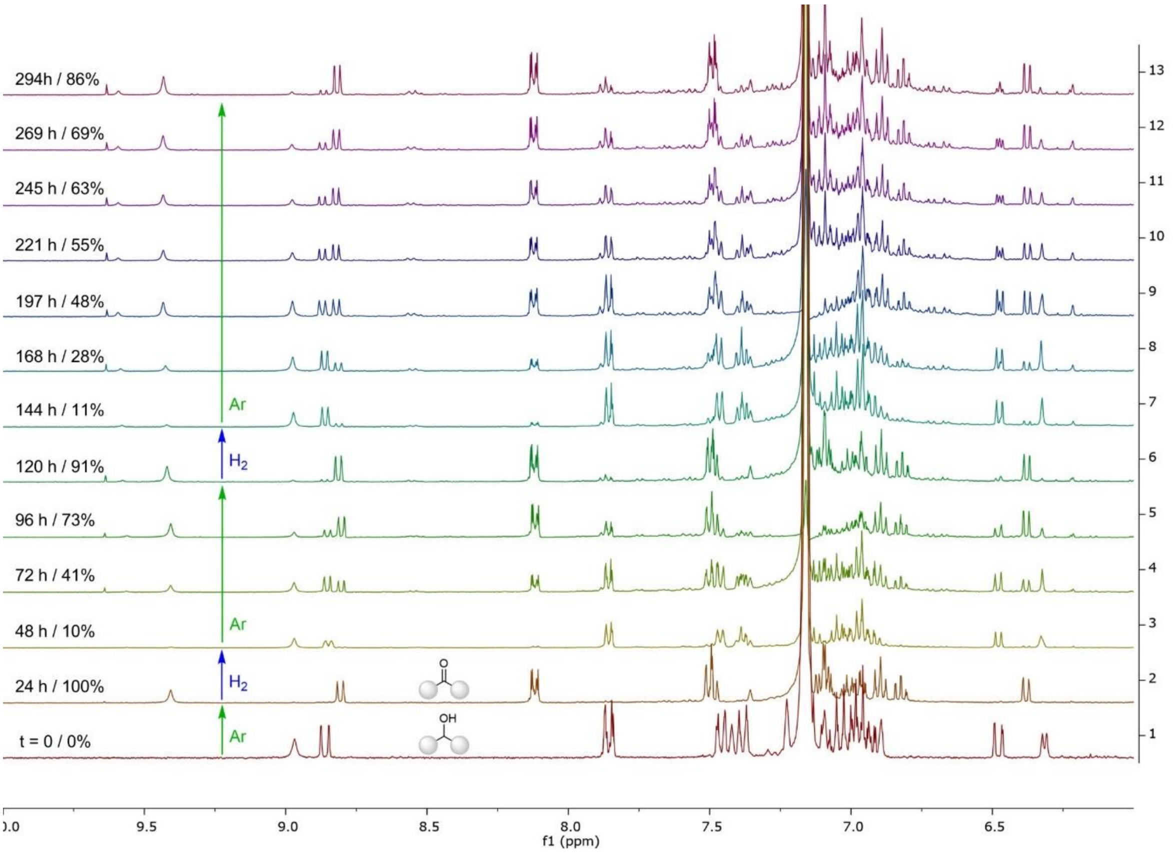

Figure S2: *^{1}H NMR monitoring of switch cycles. Experiment starts at t = 0, with 100%* ***2***$_{\textbf{\textit{alcohol}}}$ *and 0%* ***2***$_{\textbf{\textit{ketone}}}$*.*

Effect of catalyst deactivation over multiple cycles:

To a 10 mL Schlenk equipped with a cooler (3 °C) was added **2**$_{\textbf{alcohol}}$ (60.0 mg, 0.135 mmol, 1.00 equiv), **cat** (3.00 mg, 5.64 µmol, 0.04 equiv.) in C_6D_6 (6 mL). On top of the cooler were inserted in a septum 3 gas balloons (double jacked) filled with argon for the dehydrogenation process or hydrogen for the hydrogenation (the needle used was an 18 G (1.2 mm diameter)). The reaction mixture was heated at reflux (80 °C) in an oil bath (115 °C) for dehydrogenation and 70°C for hydrogenation in order to try to decrease partial switch decomposition over multiple cycles. To follow the conversion, a 0.1 mL sample of the reaction mixture is diluted with 0.4 mL of C_6D_6 and analyzed by ^{1}H NMR (**Figure S3**). When the desired conversion is obtained, the backward reaction can be set up by removing the 3 balloons and flushing the reaction mixture for 30 seconds with the other gas. Then, 3 new balloons filled with the desired gas were added. Flushing is mandatory to perform a rapid equilibrium between the gas/solution phase. After 216 h, we observe only a small amount of conversion of the alcohol to the ketone and a large amount of decomposition of the molecular switch. The iridium catalyst **cat** (3.00 mg, 5.64 µmol, 0.04 equiv.) was added again to restore the reactivity and the dehydrogenation continued under argon. Observation of the formation of the ketone confirmed the catalyst deactivation after several cycles.

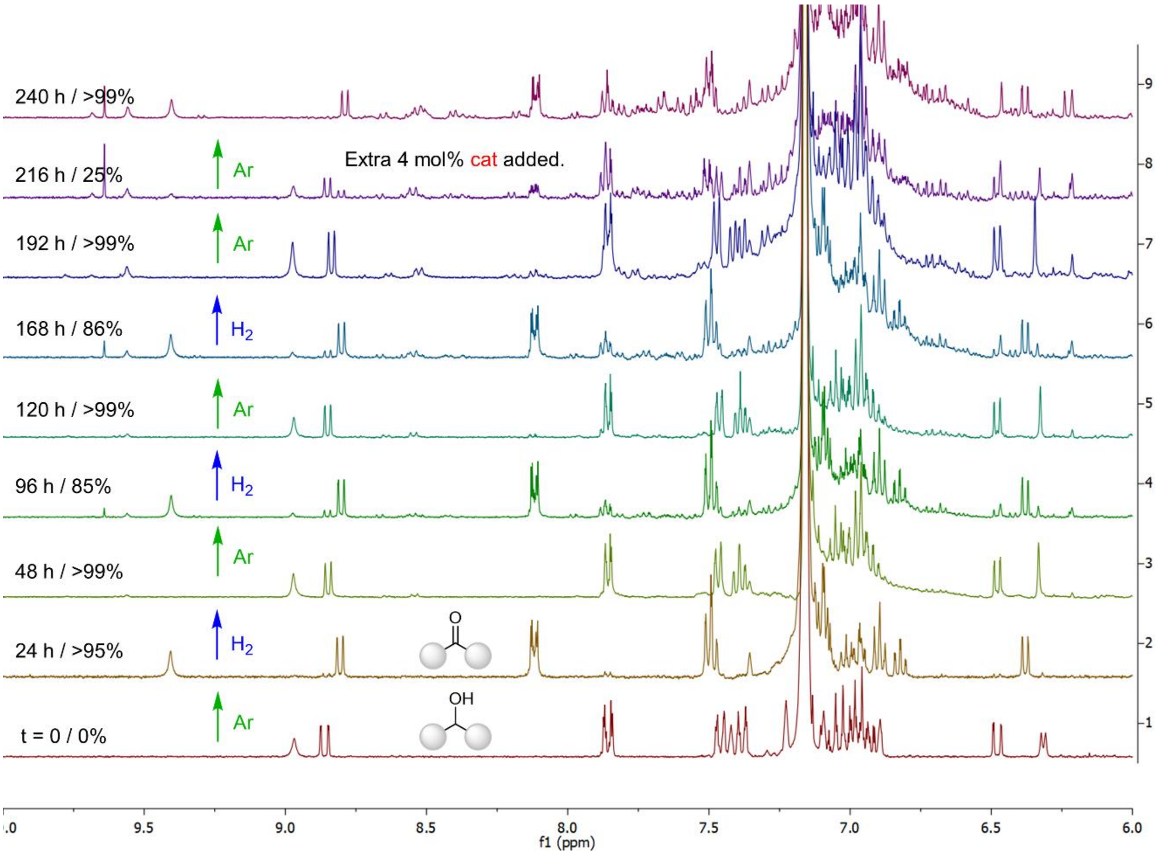

***Figure S3**: ^{1}H NMR monitoring of switch cycle and catalyst deactivation. Experiment starts at t = 0, with 100% **2**$_{alcohol}$ and 0% **2**$_{ketone}$.*

NMR Analysis

NOESY analysis of 2$_{alcohol}$ and 2$_{ketone}$:

NOESY NMR sequence were recorded in $CDCl_3$ solutions at 300K using a Bruker AVANCE III 600MHz spectrometer equipped with a Triple Resonance $^{1}H/^{13}C/^{15}N$ 5mm TXI probe with Z-gradient using Bruker Topspin 3.5.PL7 software version. The NMR sequence was *"noesygpphzs"* . The NOESY spectrum was obtained with an *F2* spectral width of 10ppm and 2K data points and an *F1* spectral width of 512 *t1* increments and 2 scans. Therefore, to reduce data acquisition time, we implemented NUS (50% random sampling). The spectra were processed by IRLS (iteratively re-weighted least squares) protocol, with SI2=4K and SI1=4k and pure cosine squared sine window functions applied to both dimensions.

On the full NOESY spectrum of $\mathbf{2_{alcohol}}$, two signals were found to be important for the conformational determination (**Figure S**). Indeed, proton **16** can perceive in its spatial environment protons **8** and proton **1**. A strong NOE signal is observed for the **16**/**8** interaction, while only a slightly distinguishable signal is observed for the **16**/**1**, confirming the predominance of the conformation where the alcohol function is on the same side as the NMe_2 moiety.

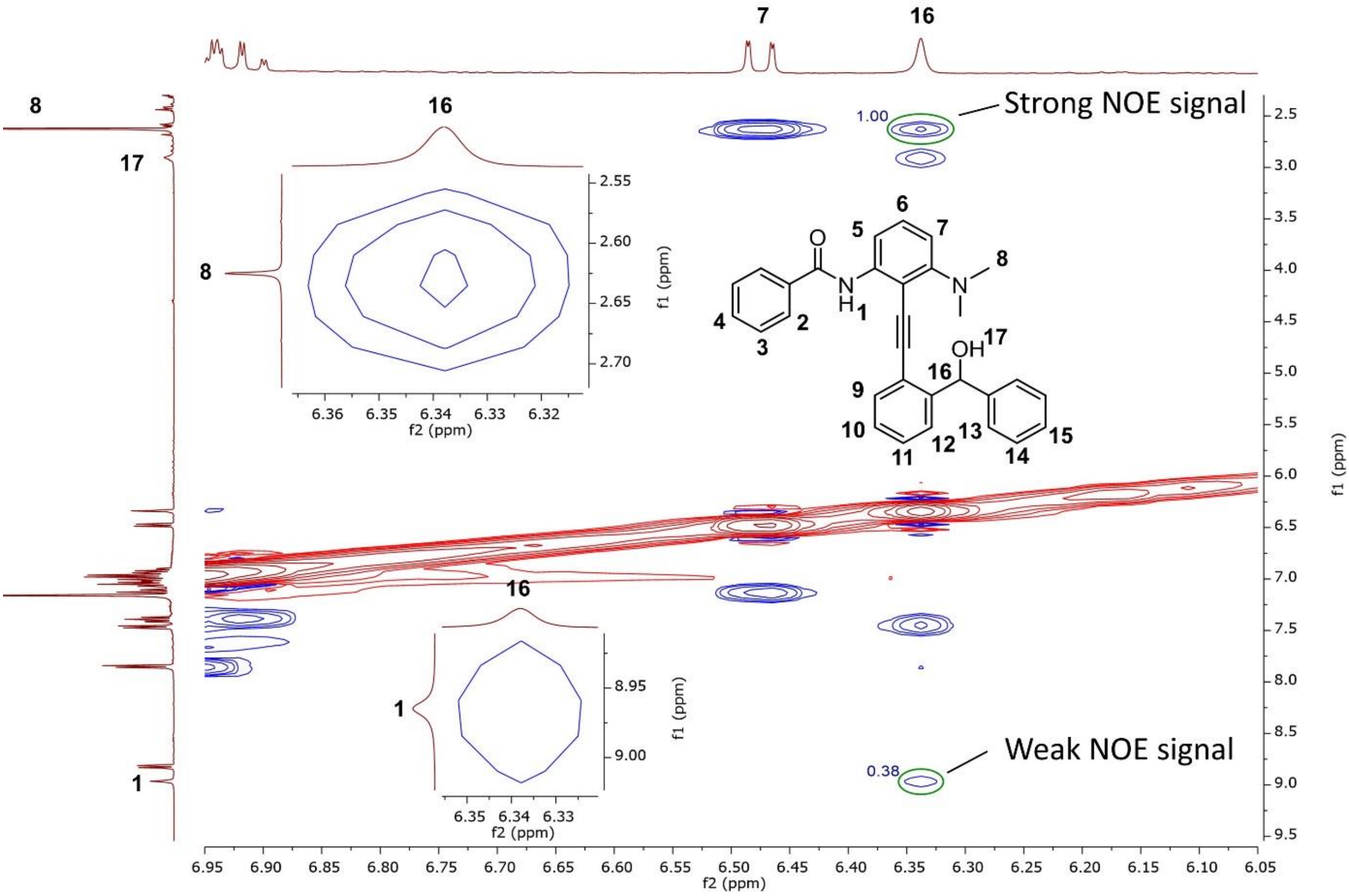

***Figure S4**: NOESY spectra of* $\mathbf{2_{alcohol}}$.

For $\mathbf{2_{ketone}}$, the NOESY spectra did not provide any additional interactions between the lower and upper part of the switch that could be used for the conformational analysis of the compound (**Figure S**).

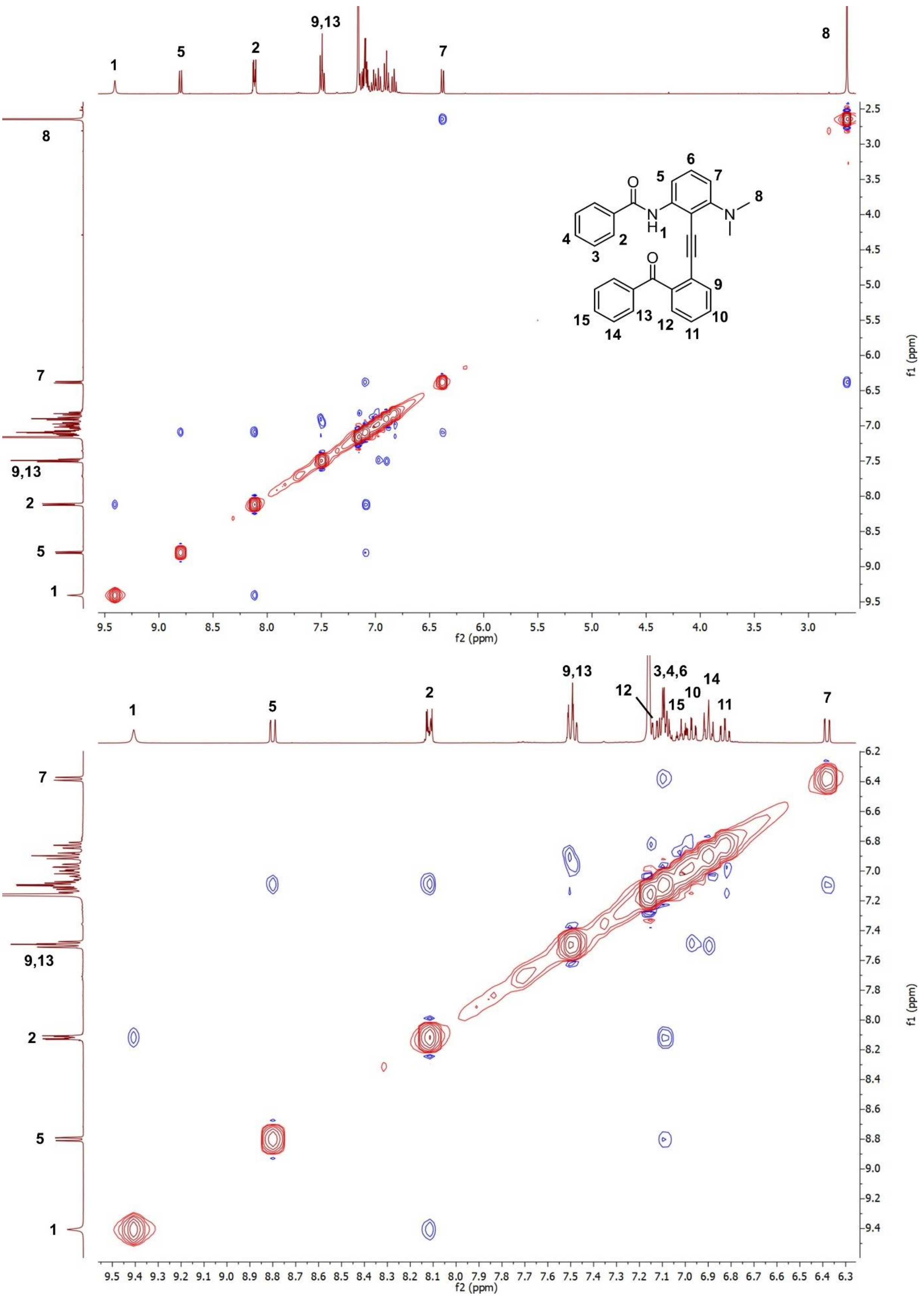

Figure S5: *NOESY spectra of* ***2**$_{ketone}$*.

NMR Analysis of enantiopure alcohols:

Enantiomers of the molecular switch **2**$_{\textbf{alcohol}}$ were obtained by chiral HPLC separation.

Sample preparation: About 35 mg of compound **2**$_{\textbf{alcohol}}$ was dissolved in 4 mL of a mixture hexane / dichloromethane /ethanol (2/1/1).

Chromatographic conditions: Chiralpak IG (250 x 10 mm), Hexane / ethanol / dichloromethane (60/10/30) as mobile phase, flow-rate = 5 mL/min, UV detection at 350 nm.

Injections (stacked): 16 times 250 mL, every 5 minutes.

Both enantiopure and racemic mixture were analyzed by ^{1}H NMR in the same condition (C_6D_6, 0.045 M, 298 K). No chemical shift was observed (**Figure S**), corresponding to the absence of a heterochiral dimeric form as the one observed in the solid state.

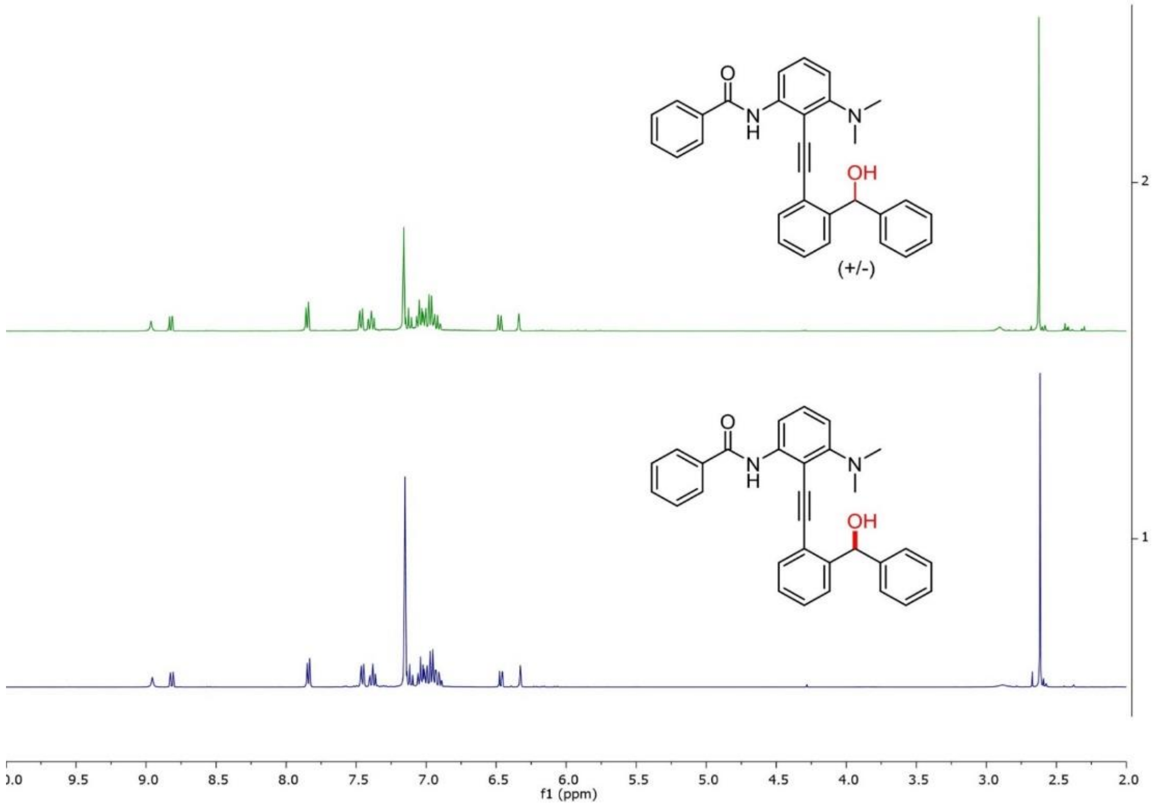

***Figure S6**: ^{1}H NMR spectra of **2**$_{\textbf{alcohol}}$. Top spectra: racemic mixture. Bottom spectra: enantiopure (<99% ee).*

NMR dilution experiments of **2**$_{\textbf{alcohol}}$ in C_6D_6 was performed and did not provide any chemical shift (**Figure S**).

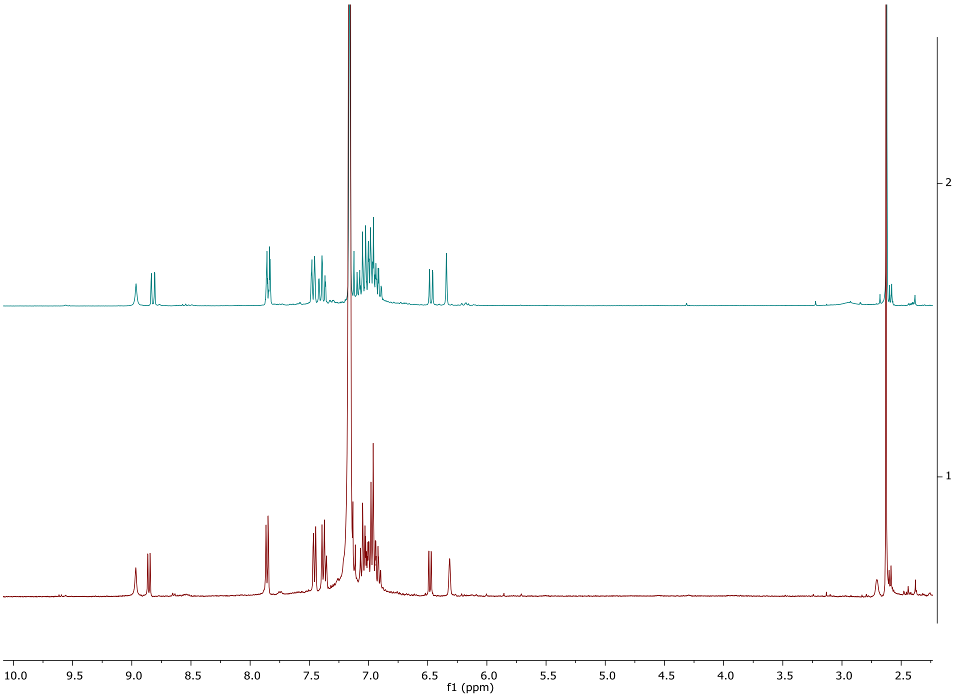

Figure S7: *^{1}H NMR spectra of* ***2*** $_{\textbf{\textit{alcohol}}}$ *at 0.045 M in* C_6D_6 *(top spectrum).* *^{1}H NMR spectra of* ***2*** $_{\textbf{\textit{alcohol}}}$ *at 0.0045 M in* C_6D_6 *(bottom spectrum).*

NMR analysis of 2_{ketone} and $2_{alcohol}$ at 300 K, 314 K and 348K.

NMR spectra were recorded on a Bruker 500 (500 MHz) spectrometer at 3 different temperatures.
In both **2**$_{\mathbf{ketone}}$ and **2**$_{\mathbf{alcohol}}$ temperature elevation has induced a partial rotation of the benzamide moiety (**Figure S**).

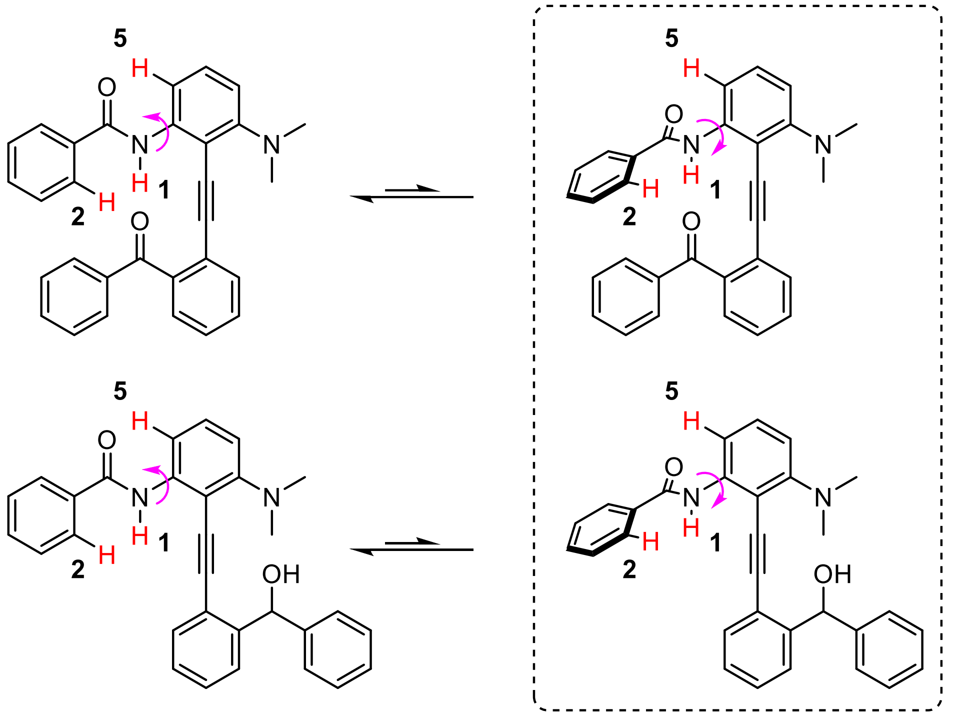

Figure S8*: Rotation of the benzamide moiety (pink arrow) lead to chemical shift on proton* ***1****;* ***2****;* ***5****.*

A similar Δδ (-0.18 ppm) was observed for proton **5** in both **2**$_{\mathbf{ketone}}$ and **2**$_{\mathbf{alcohol}}$ (**Figure S** and **Figure S**). This chemical shift has been induced from the decrease of the hydrogen bond between amide carboxyl and proton **5**. In case of proton **1** and **2** the chemical shift is different in **2**$_{\mathbf{ketone}}$ (-0.26, -0.06) and **2**$_{\mathbf{alcohol}}$ (-0.09, Ø). This result was due to the conformational difference between the two compounds. In **2**$_{\mathbf{ketone}}$, the rotation of the benzamide group decreases the hydrogen bond between ketone carbonyl and protons **1/2**. In the case of **2**$_{\mathbf{alcohol}}$ the alcohol moiety is located on the opposite side from proton **1** and **2**, leading to no supramolecular interaction.
These temperature variable experiments support the conformation switch between **2**$_{\mathbf{ketone}}$ and **2**$_{\mathbf{alcohol}}$ at the different temperatures.

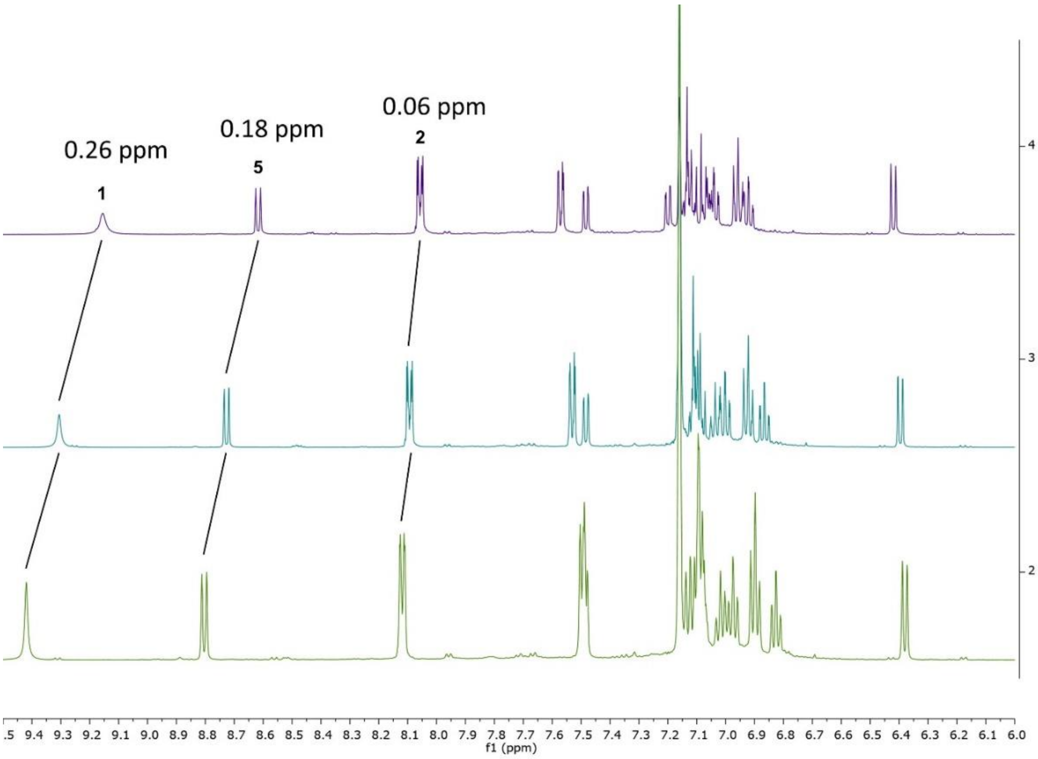

Figure S9**: ^{1}H NMR spectra of **2_{ketone} at different temperatures. **A**, 300 K (27 °C). **B**, 314 K (40 °C), **C**, 348 K (75 °C).

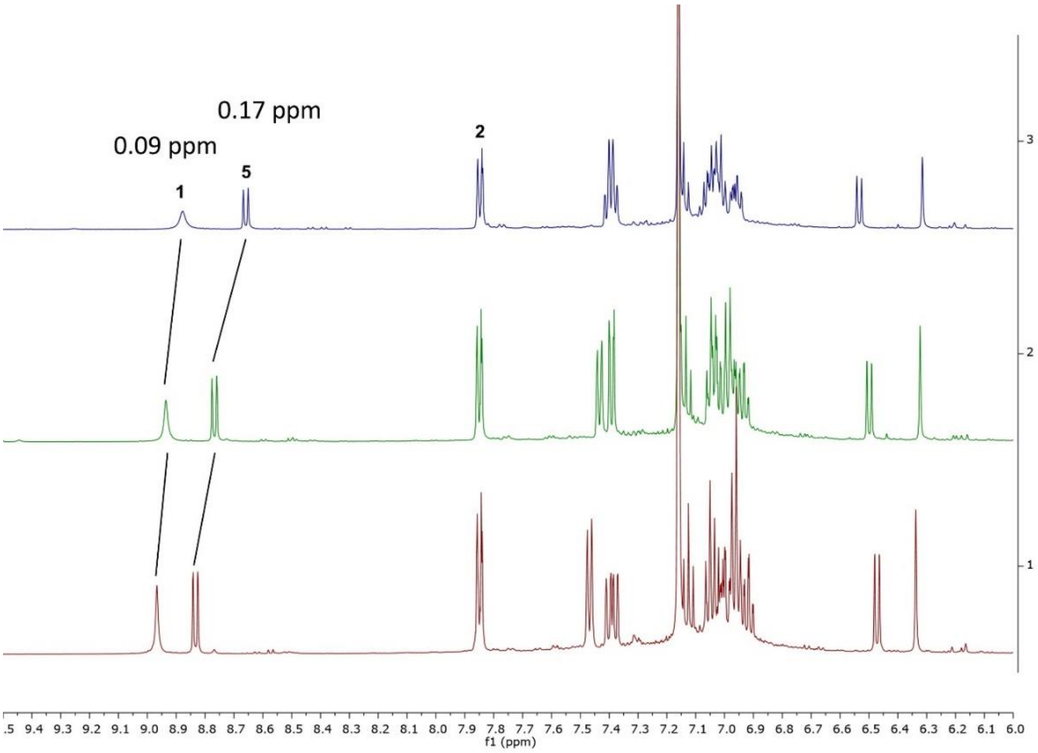

Figure S10**: ^{1}H NMR spectra of **$2_{alcohol}$ at different temperatures. **A**, 300 K (27 °C). **B**, 314 K (40 °C), **C**, 348 K (75 °C).

Tolanes ^{1}H NMR in the literature

The chemical shift of benzamide N*H* in tolane structures has already been investigated by the group of Hamilton.[4,11] This N*H* proton is prompt to interact through hydrogen bonding with a carbonyl function like an ester in the bottom part of the molecule. The resulting interaction provides a downfield move of the N*H* chemical shift in ^{1}H NMR. For example, comparison between **B** and **C** show a downfield shift around 0.5 ppm through coordination with the ester. Since in Hamilton work, competition existed between interactions with the two amides functions located on the upper part of the switch, ratio of conformers were assessed with different control amide protons (Figure S11).

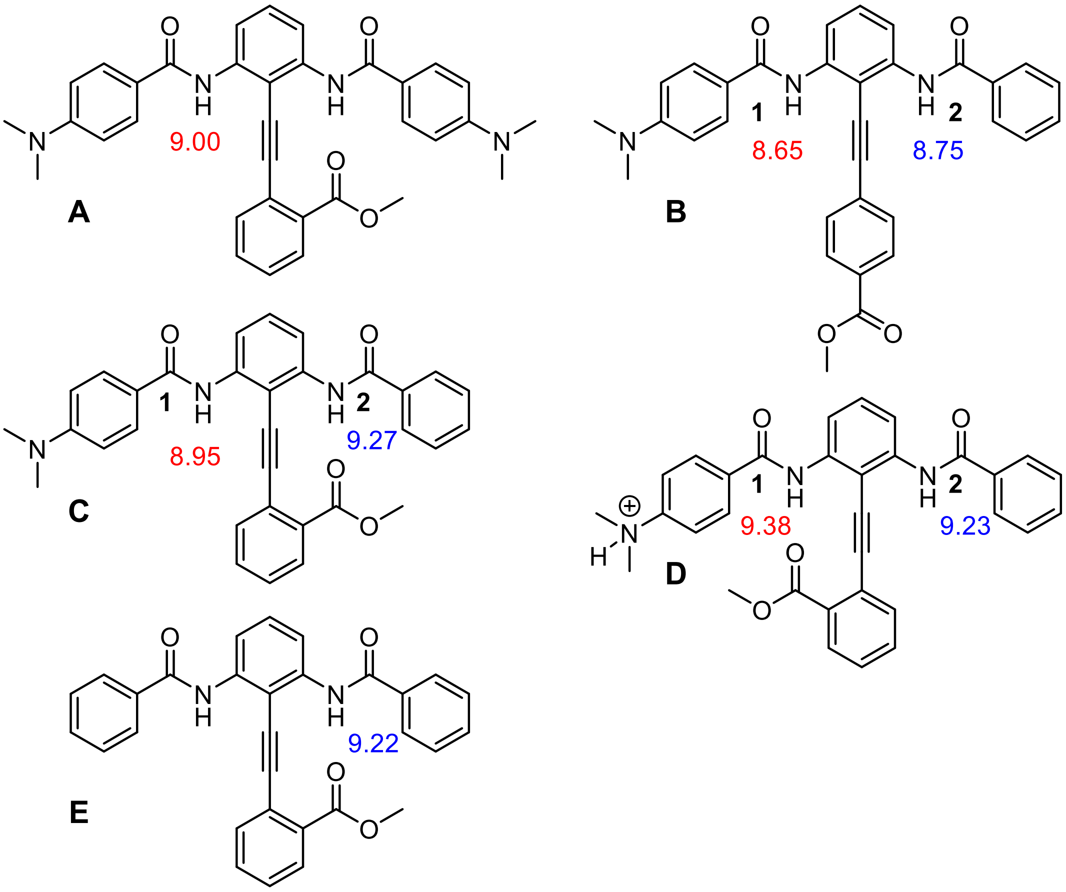

Figure S11*: Different NH chemical shift from previously reported tolanes molecules.*

Single crystal X-Ray analysis

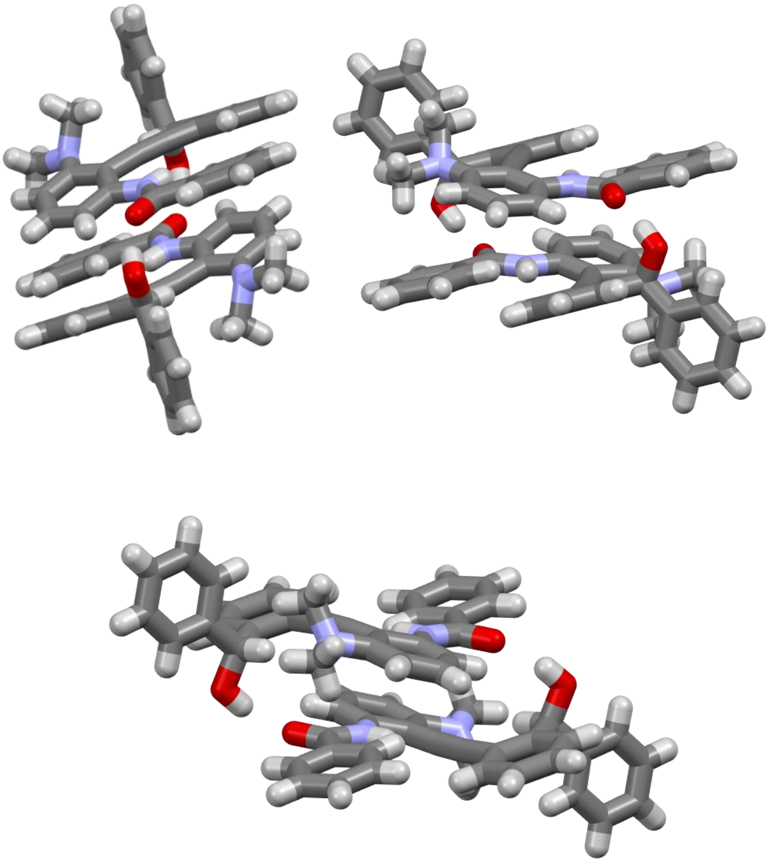

***Figure S12**: Different views from single crystal X-Ray analysis of $\mathbf{2}_{\mathbf{alcohol}}$ (dimeric form).*

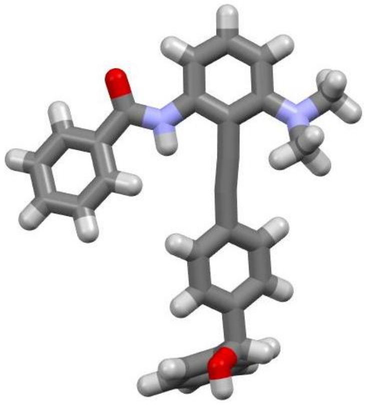

***Figure S13**: single crystal X-Ray analysis of $\mathbf{3}_{\mathbf{alcohol}}$ (monomer).*

IR analysis and Theoretical calculations

Experimental details

The infrared (IR) spectra were measured on a Bruker VERTEX70 FTIR spectrometer. A transmission cell equipped with BaF2 windows and of 200 µm of optical pathlength was used. Solutions were prepared by dissolving the solid samples in CD_2Cl_2 or in C_6D_6. Each spectrum was recorded using 30 scans with a scan frequency of 10KHz. The apodization function used is Blackman-Harris 3 terms. The spectrometer is continuously purged with dry CO_2-free air. The cell filled with solvent served as a reference. The temperature has been kept constant at 298K.

Theoretical calculations

Calculations were performed on $\mathbf{2}_{\mathbf{alcohol}}$ and $\mathbf{2}_{\mathbf{ketone}}$. The conformational study was done using a stochastic exploration of the potential energy surface (PES) using simulated annealing with RM1 semi-empirical level as implemented in Ampac10.[12] For each molecule, a set of almost 40 geometries of conformations were generated with various simulated annealing, each done with a different geometry or parameterized with a different initial temperature. A geometry optimized with SMD(C_6D_6)/B3LYP/6-311++G(d,p) level has been used as starting structure. Only the dihedral angles of this initial geometry were allowed to relax during the annealing, the bonds lengths and the valences angles were kept constant. Then, the conformations with energy lower than 2.5 kcal mol^{-1} compared to the lower energy conformation were kept and fully optimized. The geometry optimizations, vibrational frequencies, IR absorption and VCD intensities were calculated with Density Functional Theory (DFT) using B3LYP functional combined with 6-311++G(d,p) basis set. The average solvent effects were modeled implicitly by the dielectric continuum model SMD implemented in Gaussian 16.[13]
The vibrational frequencies and IR absorption intensities were calculated using the same theoretical level as for geometry optimization SMD(C_6D_6 or CD_2Cl_2)/B3LYP/6-311G++(d,p). Computed harmonic frequencies are generally larger than those experimentally observed. They have been calibrated using a standard scaling factor of 0.98. IR absorption and VCD averaged spectra were constructed from calculated dipole and rotational strengths assuming Lorentzian band shape with a half-width at half maximum of 8 cm^{-1}.
All calculations were performed using Gaussian 16 package.[13]

Analysis of $2_{alcohol}$:

Solvent effect on $2_{alcohol}$

IR spectra of **2**$_{alcohol}$ were measured in two solvents: C_6D_6 and CD_2Cl_2 (Figure S14).

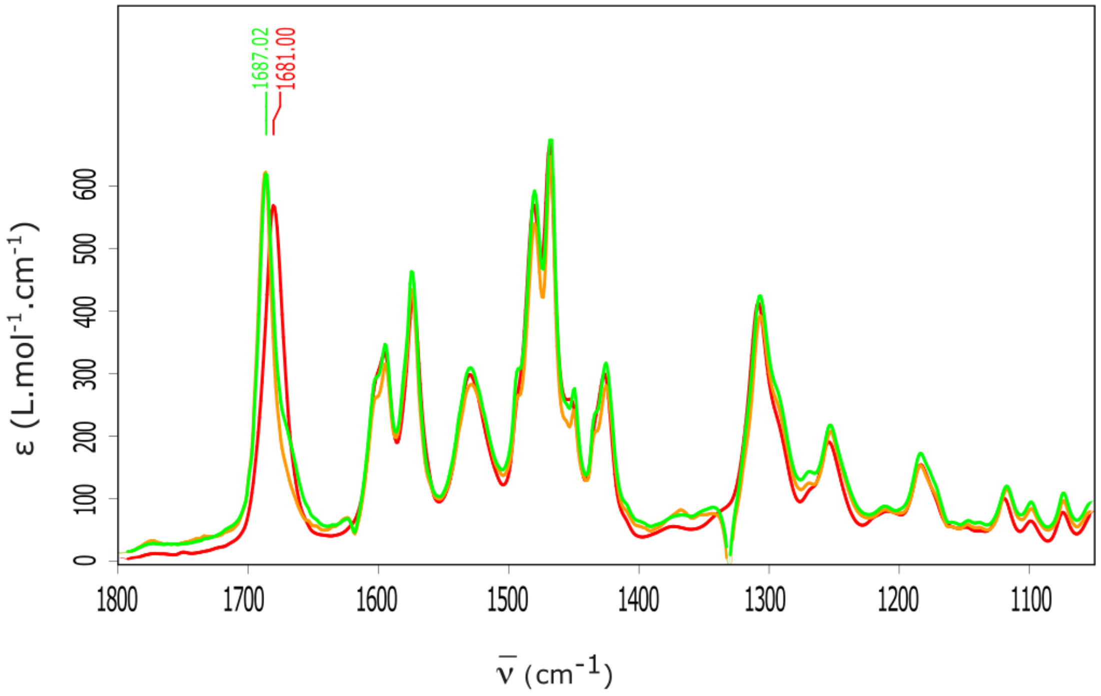

***Figure S14**: IR spectra of **2**$_{alcohol}$ racemic mixture measured in C_6D_6 (green, c= 0.08 mol.L^{-1}) and for 2nd eluted enantiomer in C_6D_6 (orange, c= 0.03 mol.L^{-1}) and in CD_2Cl_2 (red, c=0.06 mol.L^{-1})*

The C=O stretching band shifts by 6 cm^{-1} to the far infrared region when the solvent changes from C_6D_6 to CD_2Cl_2. This shift is mostly due to the difference in polarity between these two solvents.

On the IR spectrum of the racemic mixture in C_6D_6, a shoulder is observed at 1675 cm^{-1}. This shoulder is less intense for the enantiopure molecule in C_6D_6 and disappears in CD_2Cl_2. Two hypotheses can explain the presence of this additional stretching C=O band: a conformational effect (i.e there are at least two populated conformations for which the C=O group is in sufficiently different environments to induce a shift in the vibration frequency) or the presence of dimers in equilibrium with the free molecule.

Concentration effect and DFT computations

On figure S15 are displayed the IR spectra of the racemic mixture measured in C_6D_6 for various concentrations obtained by successive dilutions of a primary solution of concentration 0.1 mol.L^{-1}.

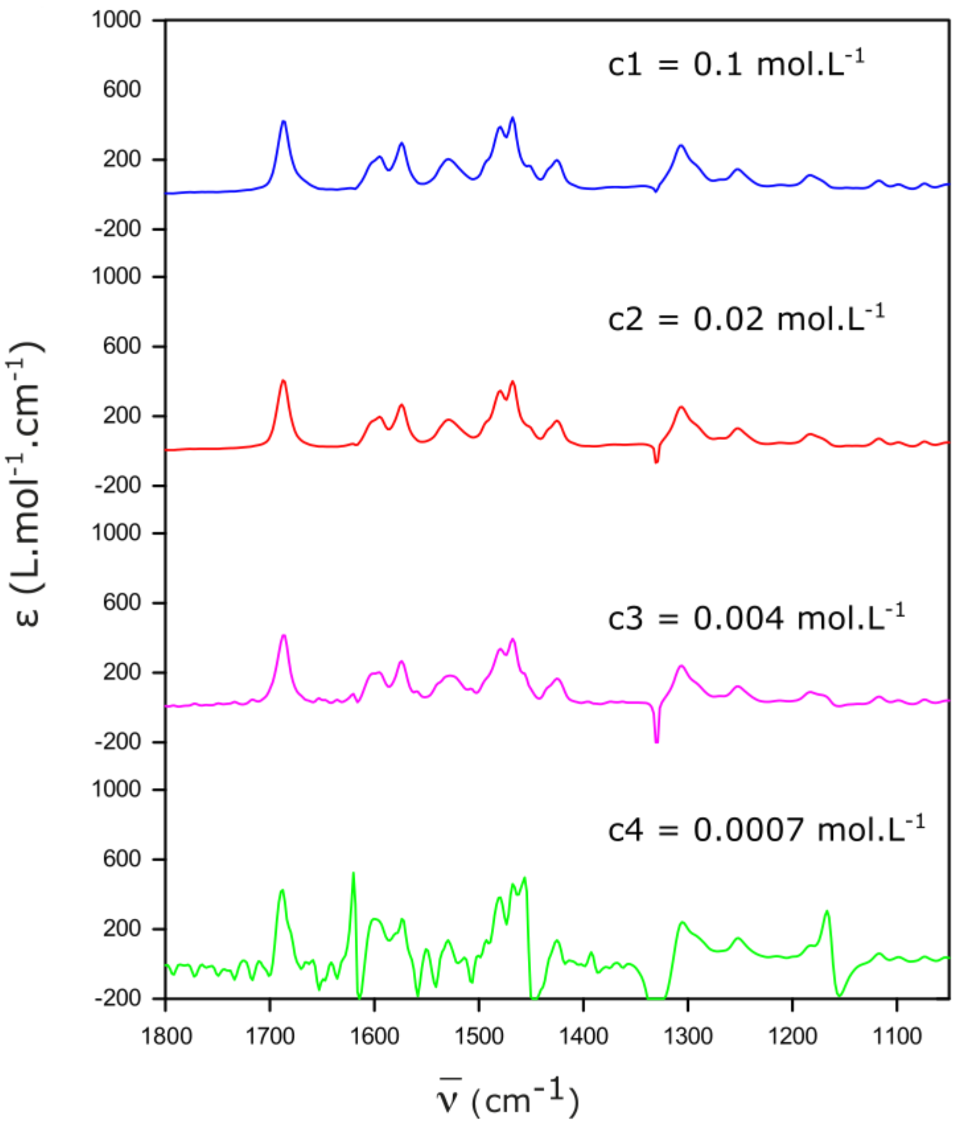

Figure S15: *IR spectra of* ***2****$_{alcohol}$ measured in C_6D_6 for successive dilutions of a primary solution: C1= 0.1mol.L^{-1}(blue), : C2= 0.02mol.L^{-1}(red), : C3= 0.004mol.L^{-1}(pink) and : C4= 0.0007mol.L^{-1}(green)*

In this concentration range, no significant modification of the spectra is observed. This result further confirms that the shoulder observed on the spectra measured in C_6D_6 would result from a conformational effect. The conformational analysis of the free molecule carried out in DFT in both solvents C_6D_6 and CD_2Cl_2 leads to the same two major conformations A_1 (or B_1) and A_2 (or B_2) (Tables S2 and S3, Figure S16). In CD_2Cl_2, there are also two other minor conformations A_3 and A_4.

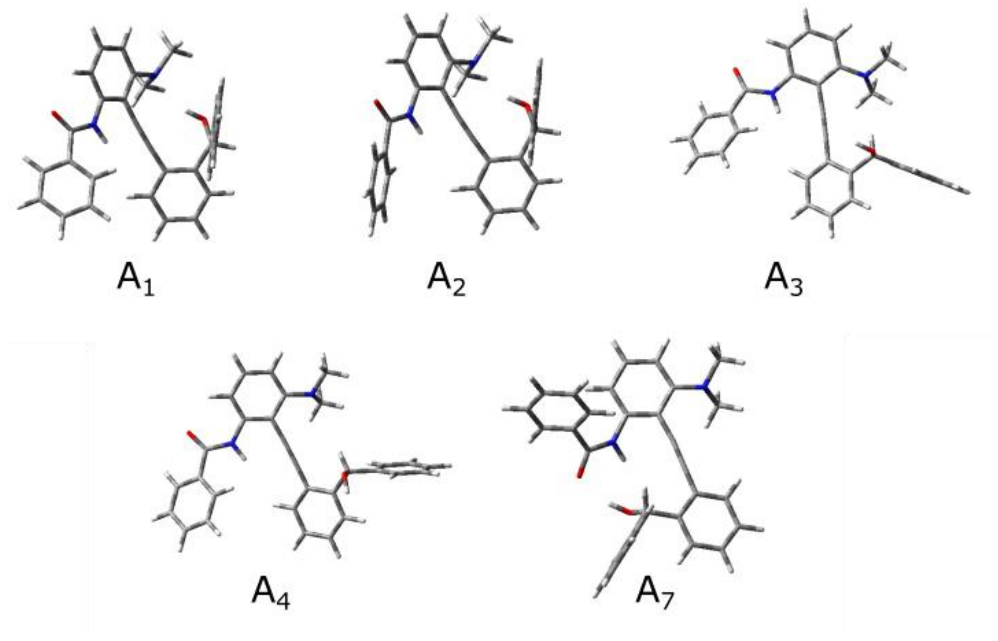

Figure S16. *Geometries of conformations A_1, A_2, A_3, A_4 and A_7 of* ***$2_{alcohol}$*** *calculated using SMD(CD_2Cl_2)/B3LYP/6-311++G(d,p) level*

Table S2 Enthalpies and Boltzmann populations of conformations A_1-A_{14} of ***$2_{alcohol}$*** *calculated using SMD(CD_2Cl_2)/B3LYP/6-311++G(d,p) level*

Conformations	H^{298K} (in a.u)	ΔH^{298K} (in kcal.mol^{-1})	Boltzmann Distribution
**A_1**	-1418.662803	0.0	**0.40**
**A_2**	-1418.662509	0.2	**0.30**
**A_3**	-1418.661937	0.5	**0.16**
**A_4**	-1418.661106	1.1	**0.07**
A_5	-1418.660664	1.3	0.04
A_6	-1418.660139	1.7	0.02
A_7	-1418.658128	2.9	0.00
A_8	-1418.656528	3.9	0.00
A_9	-1418.656382	4.0	0.00
A_{10}	-1418.655464	4.6	0.00
A_{11}	-1418.6554	4.6	0.00
A_{12}	-1418.653935	5.6	0.00
A_{13}	-1418.6537	5.7	0.00

A_{14}	-1418.651032	7.4	0.00

*Table S3 Enthalpies and Boltzmann populations of conformations B_1-B_{13} of **2**$_{\textbf{alcohol}}$ calculated using SMD(C_6D_6)/B3LYP/6-311++G(d,p) level*

Conformations	H^{298K} (in a.u)	ΔH^{298K} (in kcal.mol^{-1})	Boltzmann Distribution
B_1	-1418.654499	0.0	**0.58**
B_2	-1418.65404	0.3	**0.36**
B_3	-1418.651423	1.9	0.02
B_4	-1418.65093	2.2	0.01
B_5	-1418.650857	2.3	0.01
B_6	-1418.650772	2.3	0.01
B_7	-1418.649568	3.1	0.00
B_8	-1418.648063	4.0	0.00
B_9	-1418.646581	5.0	0.00
B_{10}	-1418.64645	5.1	0.00
B_{11}	-1418.645252	5.8	0.00
B_{12}	-1418.643691	6.8	0.00
B_{13}	-1418.640387	8.9	0.00

The conformations A_1 (or B_1) and A_2 (or B_2) are geometrically very close and owe their greater stability to the synergy of several stabilizing interactions (OH...NMe_2 hydrogen bonding, π-stacking and Van der Walls interactions). Among the conformations found in our analysis, we find that those for which there is hydrogen bonding between the alcohol and amide functions (A_7 for instance) have a calculated enthalpy much higher than that of the most stable conformation and are therefore not populated (figure S15 and table S2). Regarding these results, a conformational effect could only occur from an explicit interaction between the solute and the solvent (this effect not being taken into account in the theoretical SMD model used which treats the solvent implicitly). If we cannot exclude this possibility, the comparison of the measured and calculated spectra is more in line with the hypothesis of an equilibrium between free molecules and dimers. Indeed, the resulting average spectra calculated in CD_2Cl_2 and in C_6D_6 are similar and consistent with the experimental measurements. In particular, the solvent effect observed experimentally at the C=O elongation band is correctly predicted by the calculations (Figures S17 and S2).

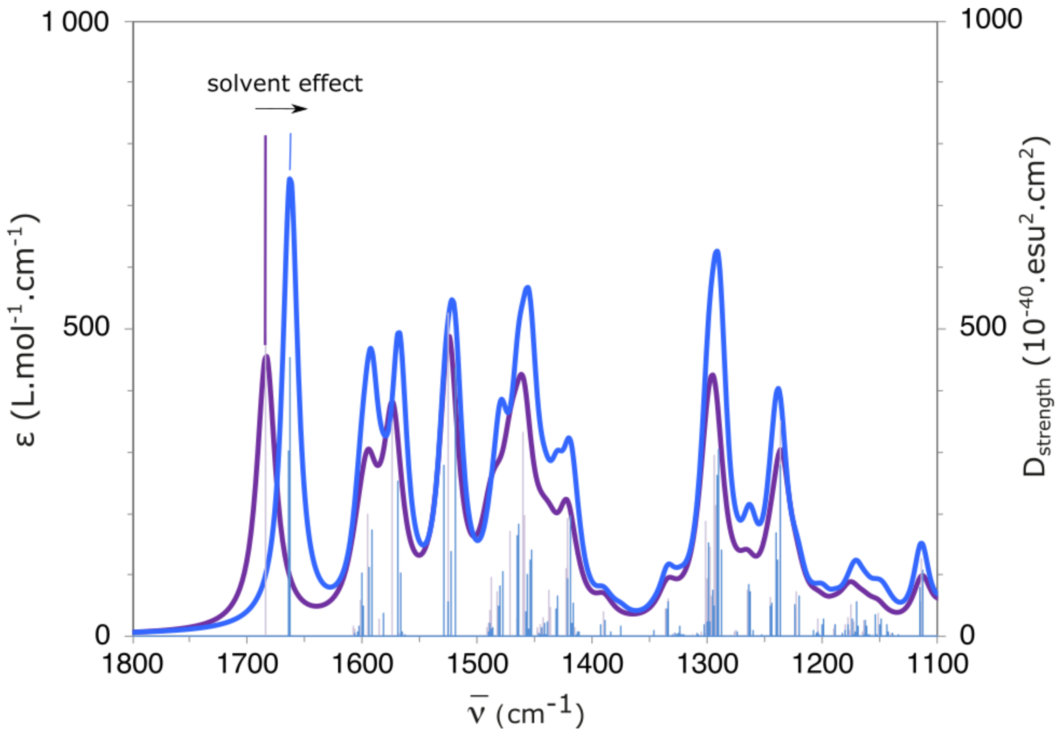

Figure S17. *Calculated IR spectra of **2**$_{alcohol}$ in CD_2Cl_2 (blue, with dipole strength in clear blue) and spectra in C_6D_6 (purple, with dipole strength in clear purple)*

We can see the shift towards the far IR spectral range of the C=O stretching band in CD_2Cl_2. The calculated shift is however larger than the measured one which suggests that the calculation method used seems to slightly overestimates this effect. In the spectral range 1100-2000 cm^{-1} a satisfying agreement is obtained between the measured and the calculated spectra in CD_2Cl_2 for the free molecule (figure S18). Similarly, the measured and the calculated IR spectra for the free molecule in C_6D_6 are also in good agreement (figure S19). It is therefore reasonable to conclude that the dominant conformations in solution are those in which a hydrogen bond is formed between the alcohol function and the dimethylamino moiety (figure S16).

The calculated average IR spectrum was built from the weighted sum of the spectra of the conformations that were found. The weighting coefficient is the Boltzmann population of each conformation, calculated using the enthalpies at 298 K from the DFT calculations (figure S17 and table S2).

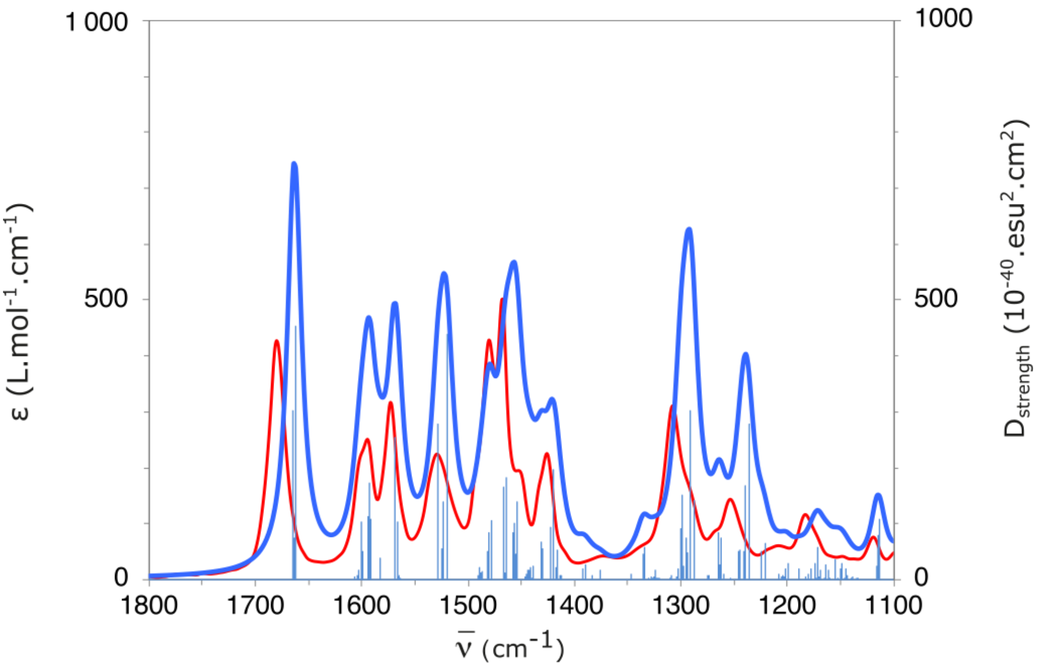

Figure S18 *Measured (red, c=0,06 mol.L^{-1}) and calculated (blue, with dipole strength in clear blue) IR spectra of **2**$_{alcohol}$ in CD_2Cl_2.*

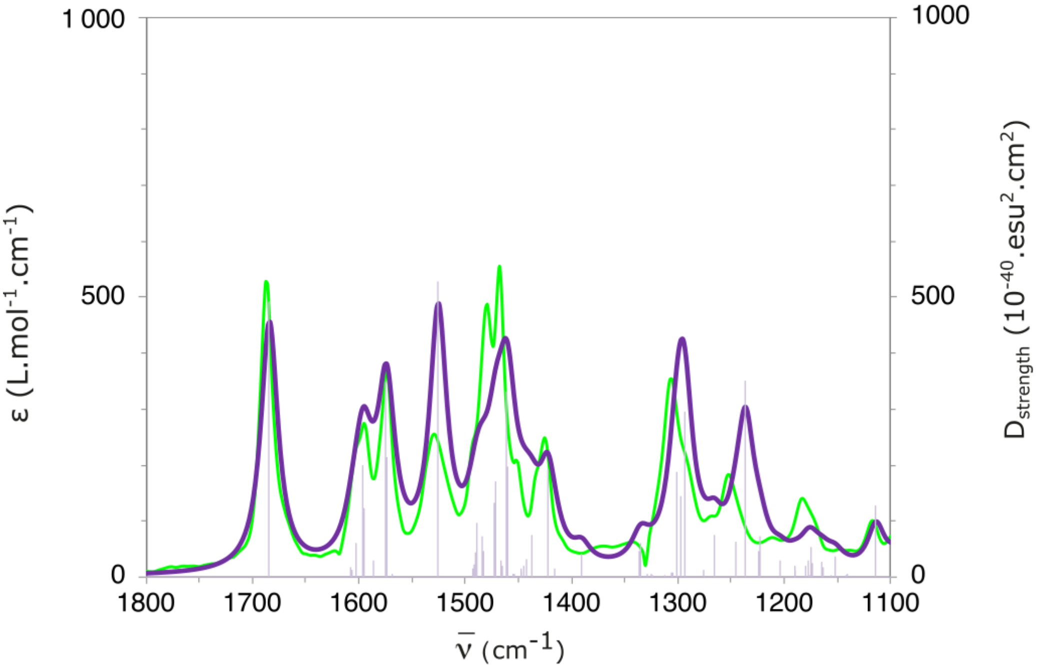

Figure S19 *Measured (green, c= 0,08 mol.L^{-1}) and calculated (purple, with dipole strength in clear purple) IR spectra of **2**$_{alcohol}$ in C_6D_6.*

This result allows us to assume that the free molecule is the predominant state in solution. Despite π-stacking interactions with a C_6D_6 molecules cannot be ruled out, it is reasonable to assume that they are less favorable than dimer formation. In order to support this hypothesis, we have optimized the

geometry of a dimer in C_6D_6, extracted from the structure obtained by single crystal X-ray diffraction (figure S18a). For these calculations the dispersion potential GD3BJ of Grimme's dispersion with Becke-Johnson damping was introduced to improve the description of the dimer. For a consistent comparison, GD3BJ was also introduced for the calculation of the free molecule spectrum. The comparison of the IR spectrum calculated for this dimer geometry with the one calculated for the free molecule (figure S18b) shows that in the presence of an equilibrium between these two forms of the molecule and in the hypothesis of an equilibrium shifted to the free molecule, only the position of the C=O elongation band would distinguish them. This is consistent with the experimental observation. Due to the polarity of dichloromethane, the formation of dimer is less favorable than in C_6D_6, which is what we observe on the IR spectra measured in this solvent.

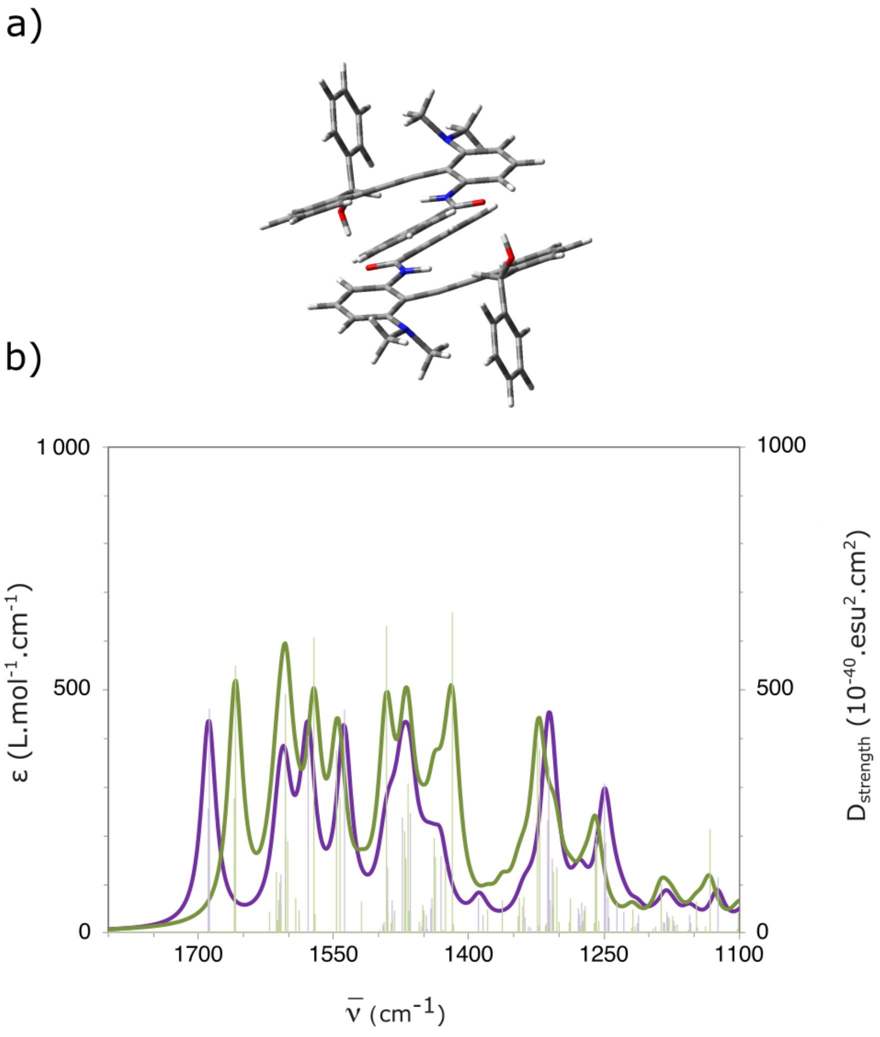

***Figure 20**. a)- Geometry of dimer of **$2_{alcohol}$** optimized using SMD(C6D6)/GD3BJ-B3LYP/6-311++G(d,p) b)- Calculated IR spectra in C_6D_6 of alone molecule (purple) and of the dimer D1(dark green).*

Analysis of 2-ketone:

The same study was carried out with **2ketone** in C_6D_6. The conformational analysis of this molecule led us to calculate 13 conformations (table S4). Among these conformations, 5 have calculated Boltzmann populations higher than 5% and were thus retained to build the calculated IR spectrum (figure S21). According to the interactions between the ketone function and the benzamide moiety, we can classify these 5 conformations. Firstly, there are the conformations C_1 and C_3 characterized by a hydrogen bond between the ketone function and the NH group of the amide as well as by a CH-π type interaction between a CH of the phenyl moiety of the ketone and the π system of the benzamide moiety. The C_2 and C_4 conformations are characterized by a double interaction of the ketone function with the NH and a $C\text{-}H_{(benz.)}$ of the benzamide moiety. Finally, the least populated conformation C_5 is characterized by the fact that the ketone function has no interaction with the benzamide moiety.

Table S4 Enthalpies and Boltzmann populations of conformations C_1-C_{13} of ***2ketone*** *calculated using SMD(C6D6)/B3LYP/6-311++G(d,p) level*

Conformations	H^{298K} (in a.u)	ΔH^{298K} (in kcal.mol^{-1})	Boltzmann Distribution
**C_1**	-1417.473433	0.0	**0.33**
**C_2**	-1417.472853	0.4	**0.18**
**C_3**	-1417.472824	0.4	**0.17**
**C_4**	-1417.472414	0.6	**0.11**
**C_5**	-1417.472176	0.8	**0.09**
C_6	-1417.471613	1.1	0.05
C_7	-1417.471404	1.3	0.04
C_8	-1417.470938	1.6	0.02
C_9	-1417.470628	1.8	0.02
C_{10}	-1417.465779	4.8	0.00
C_{11}	-1417.465744	4.8	0.00
C_{12}	-1417.464794	5.4	0.00
C_{13}	-1417.464414	5.7	0.00

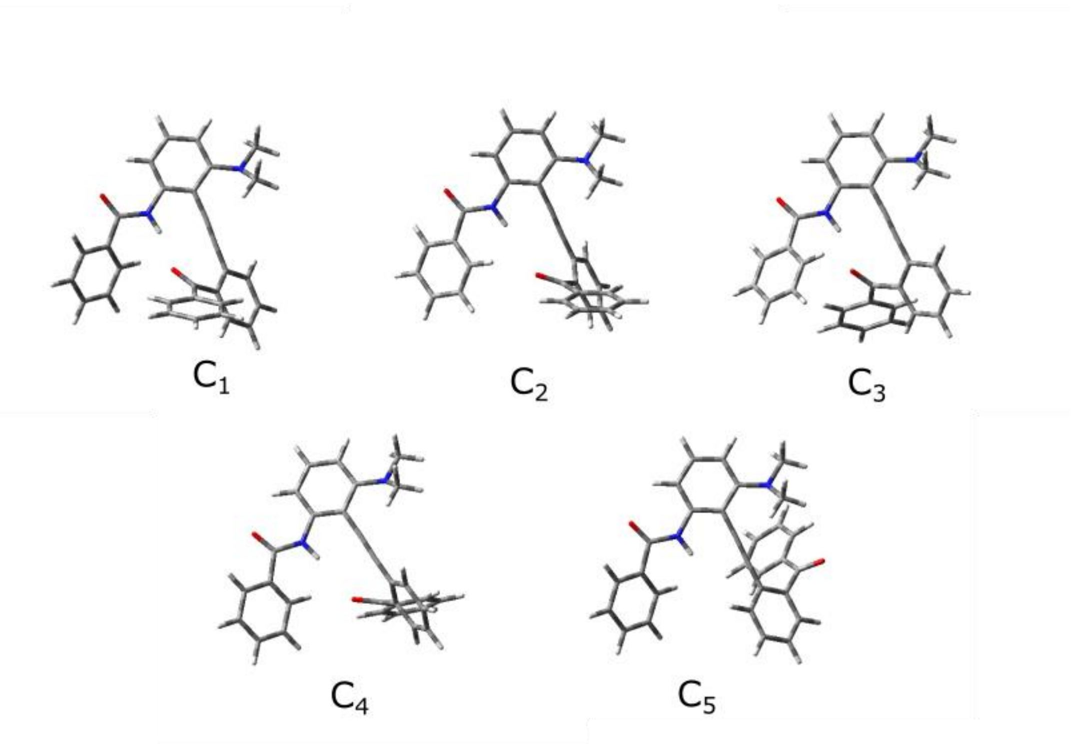

***Figure S21**. Geometries of conformations C_1, C_2, C_3, C_4 and C_5 of **2_{ketone}** calculated using SMD(C6D6)/B3LYP/6-311++G(d,p) level*

The comparison of the measured and calculated IR spectra shows a satisfactory agreement (Figure S22). As before, it is reasonable to conclude that the dominant conformations of **2_{ketone}** in C_6D_6 solution are those in which the ketone interacts with the benzamide moiety although the calculations predict a minor conformation (<1%) without this kind of interactions.

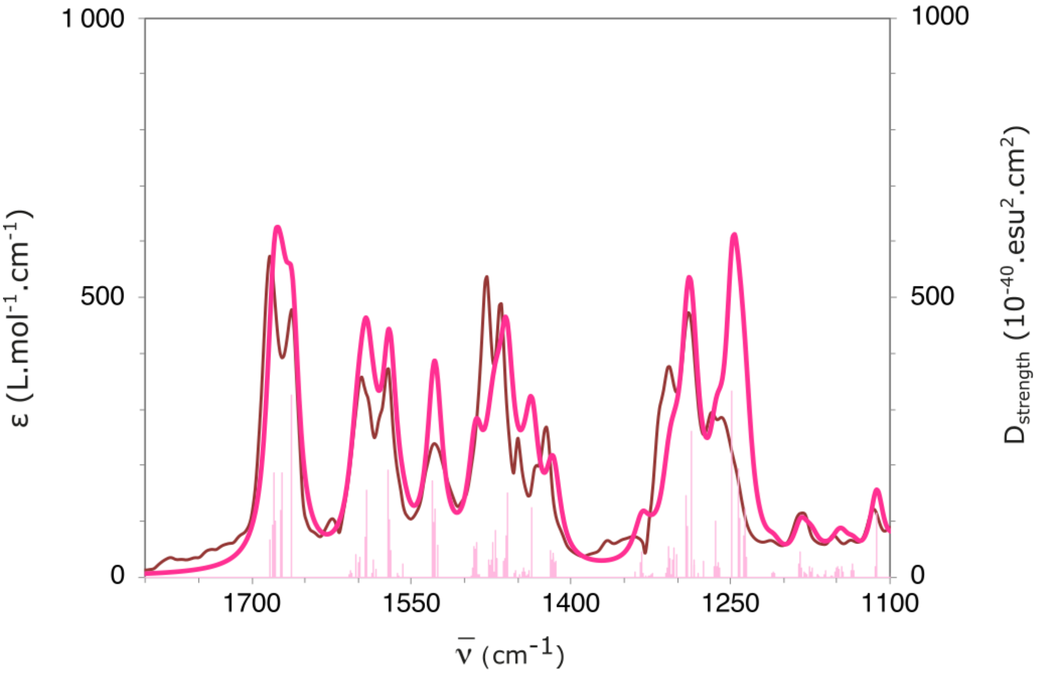

***Figure S22** Measured (brown, c=0.08mol.L^{-1}) and calculated (pink, with dipole strength in clear pink) IR spectra of **2_{ketone}** in C_6D_6.*

DFT calculation of the energy of rotation of conformers C1 and C2 of 2-ketone:

All geometries were calculated with Density Functional Theory (DFT) using B3LYP functional combined with 6-311G(d,p) basis set. The average solvent effects were modeled implicitly by the dielectric continuum model SMD implemented in Gaussian 16. The calculations were carried out in benzene. The vibrational frequencies were calculated using the same theoretical level as for geometry optimization SMD(C_6D_6)/B3LYP/6-311G(d,p). Thus, the transition state was characterized by a single imaginary frequency and the local minima by no imaginary frequency. The link between the transition state and the minima was checked by IRC calculations. The rotation barriers were evaluated from the enthalpy calculations at 298.15 K.

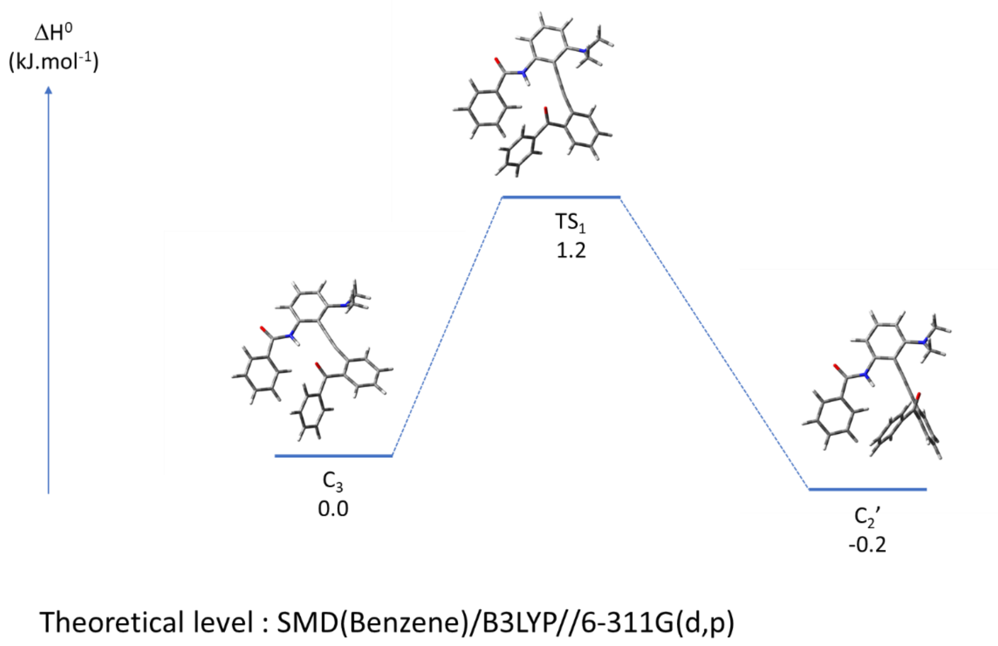

Figure S23 *Calculated transition state for the rotation of the alkyne in* $\mathbf{2_{ketone}}$ *in* C_6D_6*.*

NMR spectra

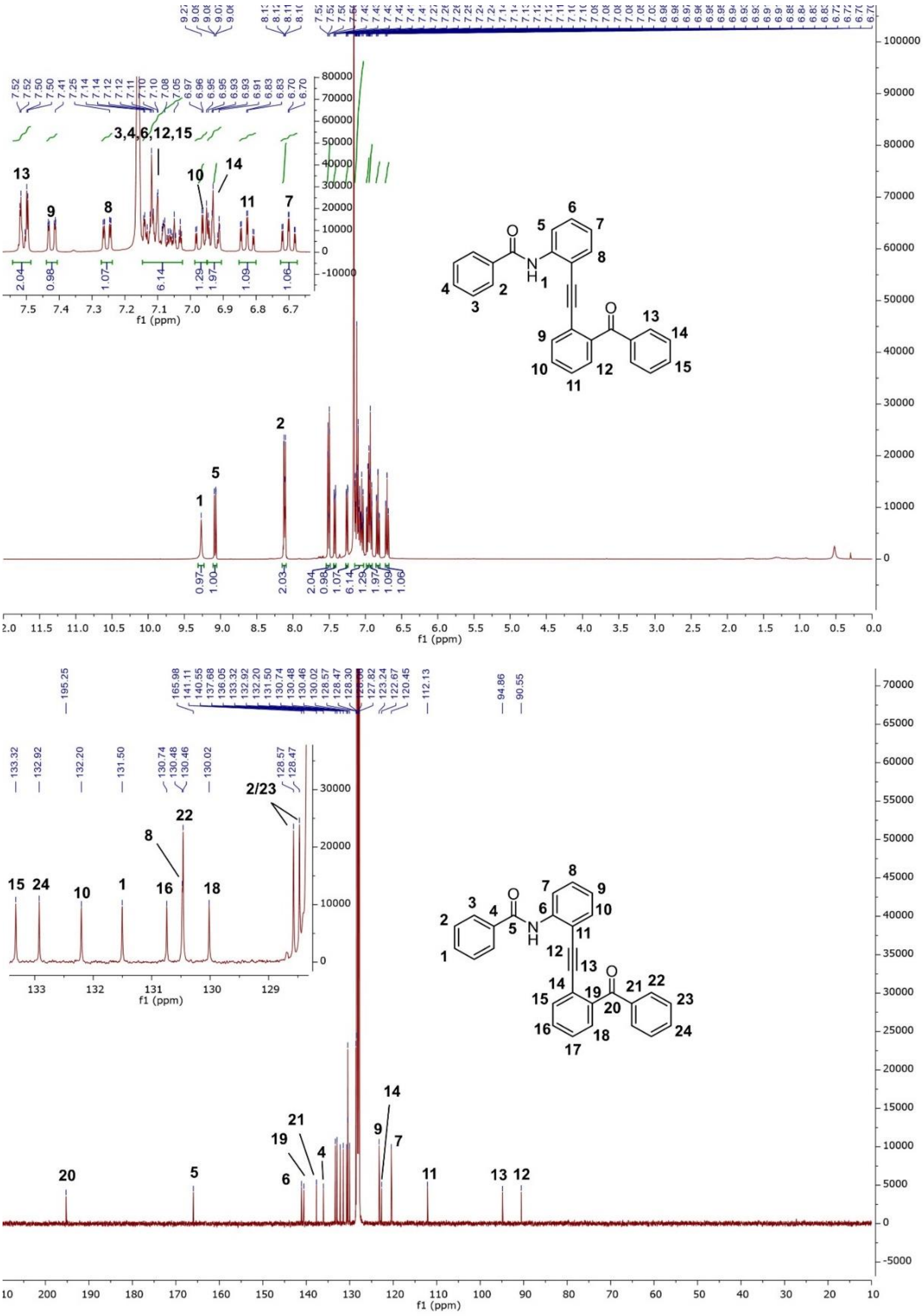

Figure S24: ^{1}H and ^{13}C NMR spectra of $\mathbf{1}_{\mathbf{\textit{ketone}}}$ at 0.045 M in C_6D_6.

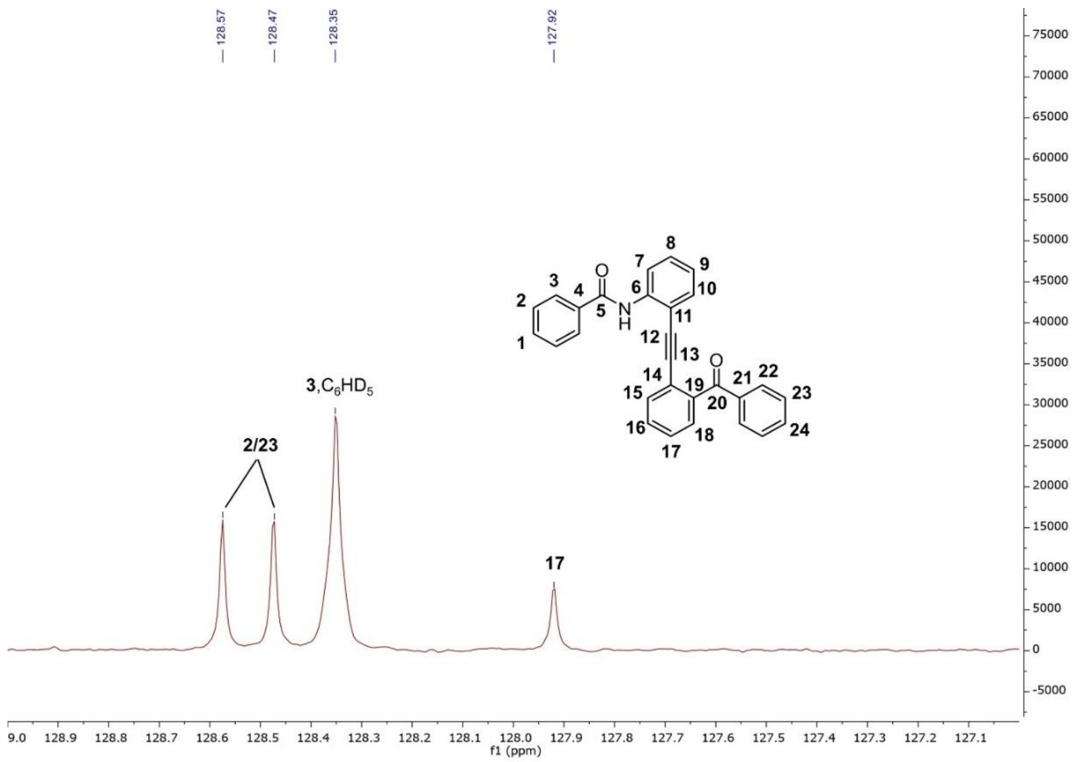

***Figure S25**: DEPT 135 NMR spectra of **1**$_{\textbf{ketone}}$ at 0.045 M in C_6D_6.*

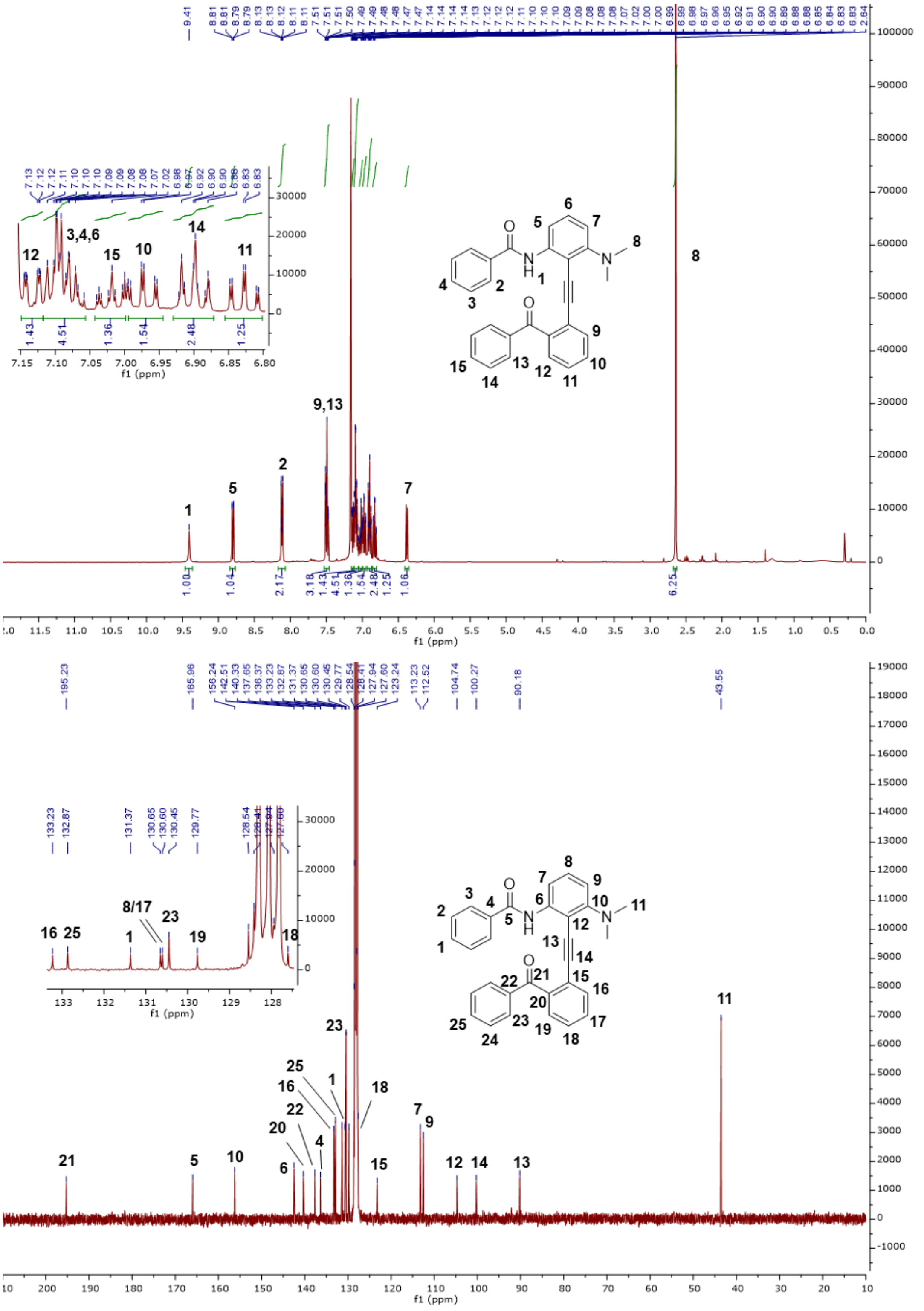

***Figure S26**: ^{1}H and ^{13}C NMR spectra of **2**$_{\mathbf{ketone}}$ at 0.045 M in C_6D_6.*

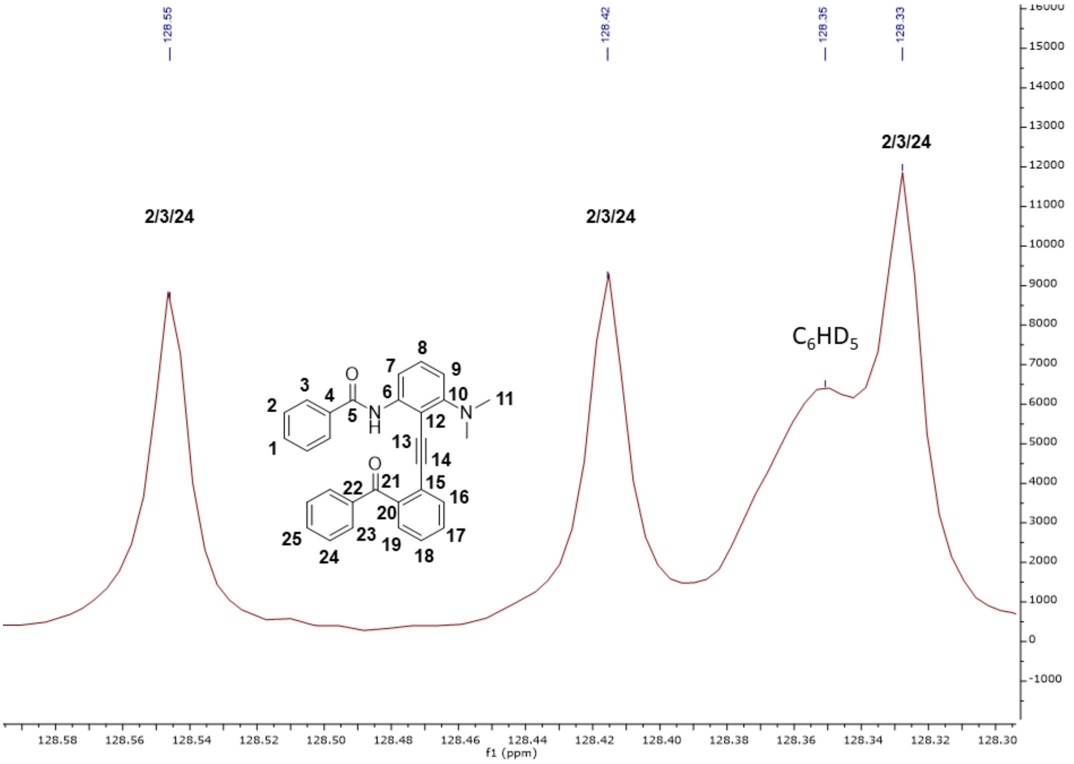

Figure S27: *DEPT 135 NMR spectra of* ***2**$_{ketone}$ *at 0.045 M in* C_6D_6*.*

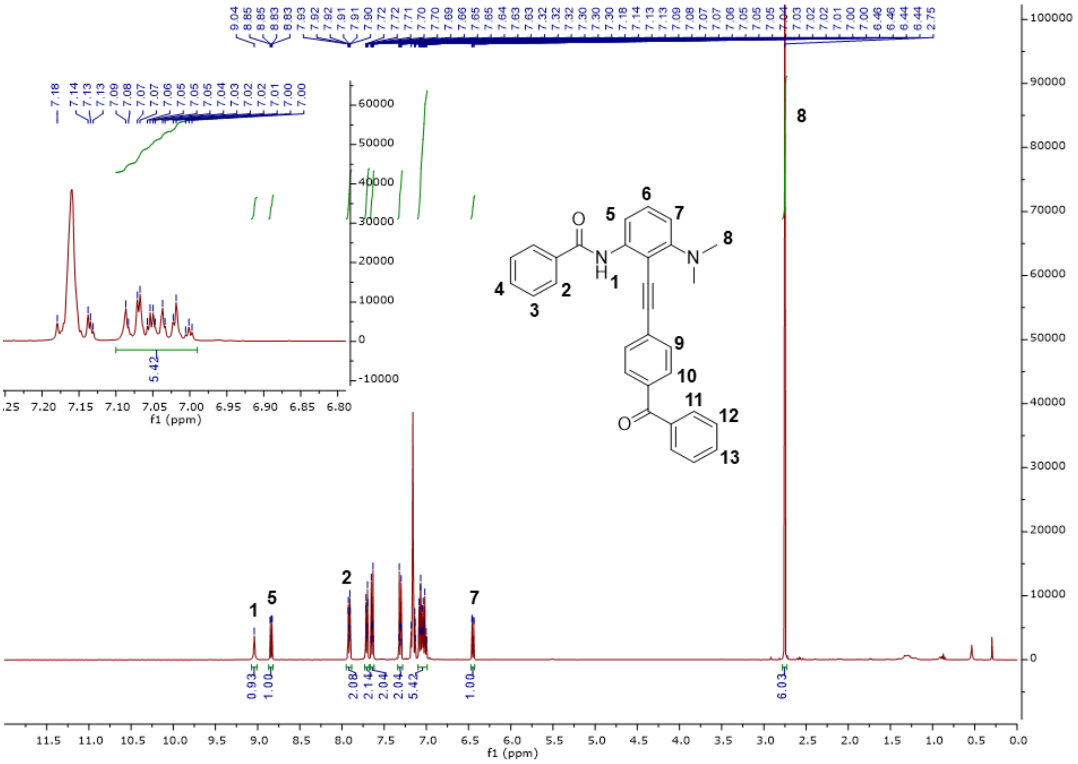

Figure S28: ^{1}*H NMR spectra of* ***3**$_{ketone}$ *at 0.045 M in* C_6D_6*.*

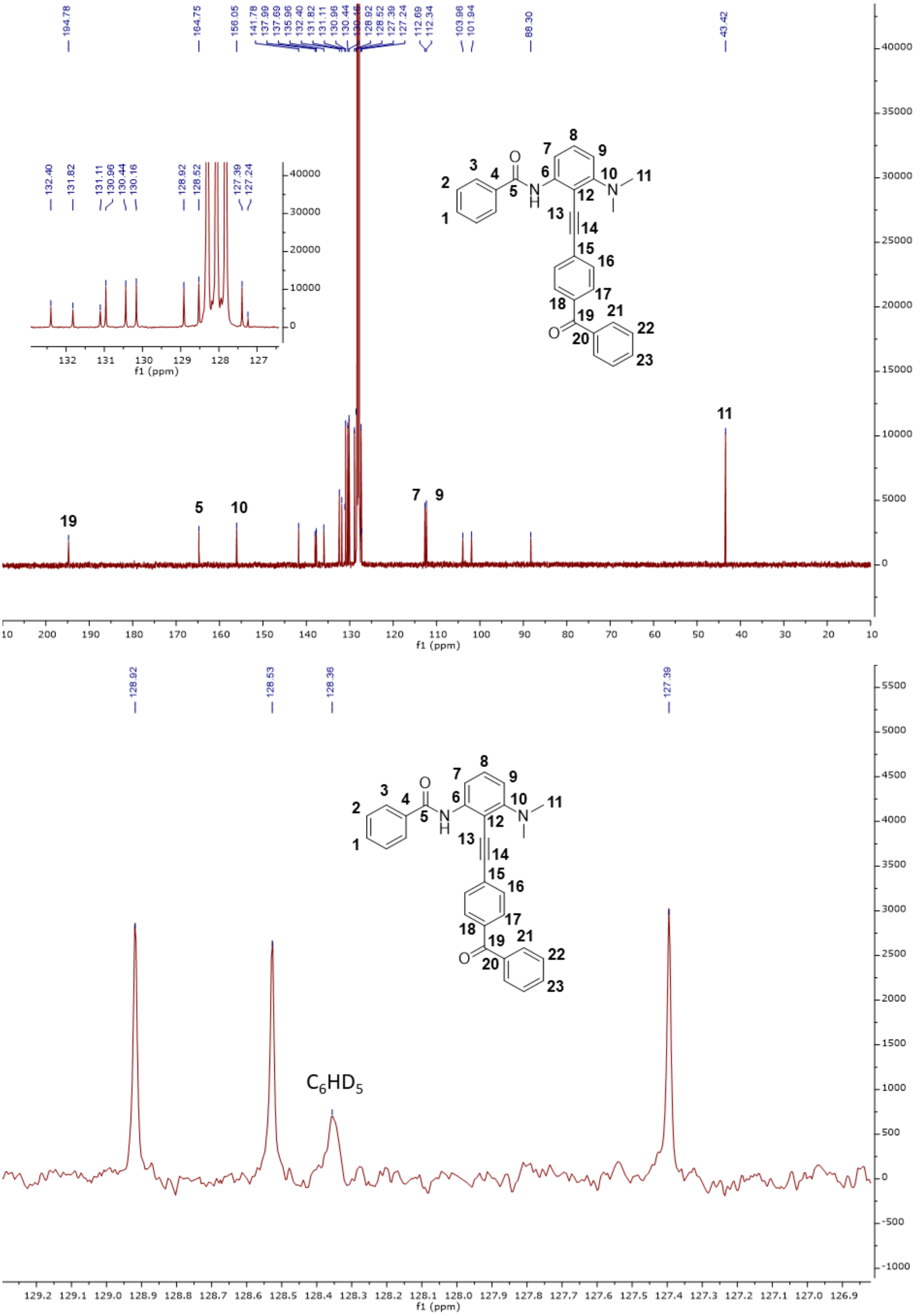

Figure S29: *^{13}C and DEPT 135 NMR spectra of* ***3**$_{ketone}$* *at 0.045 M in* C_6D_6.

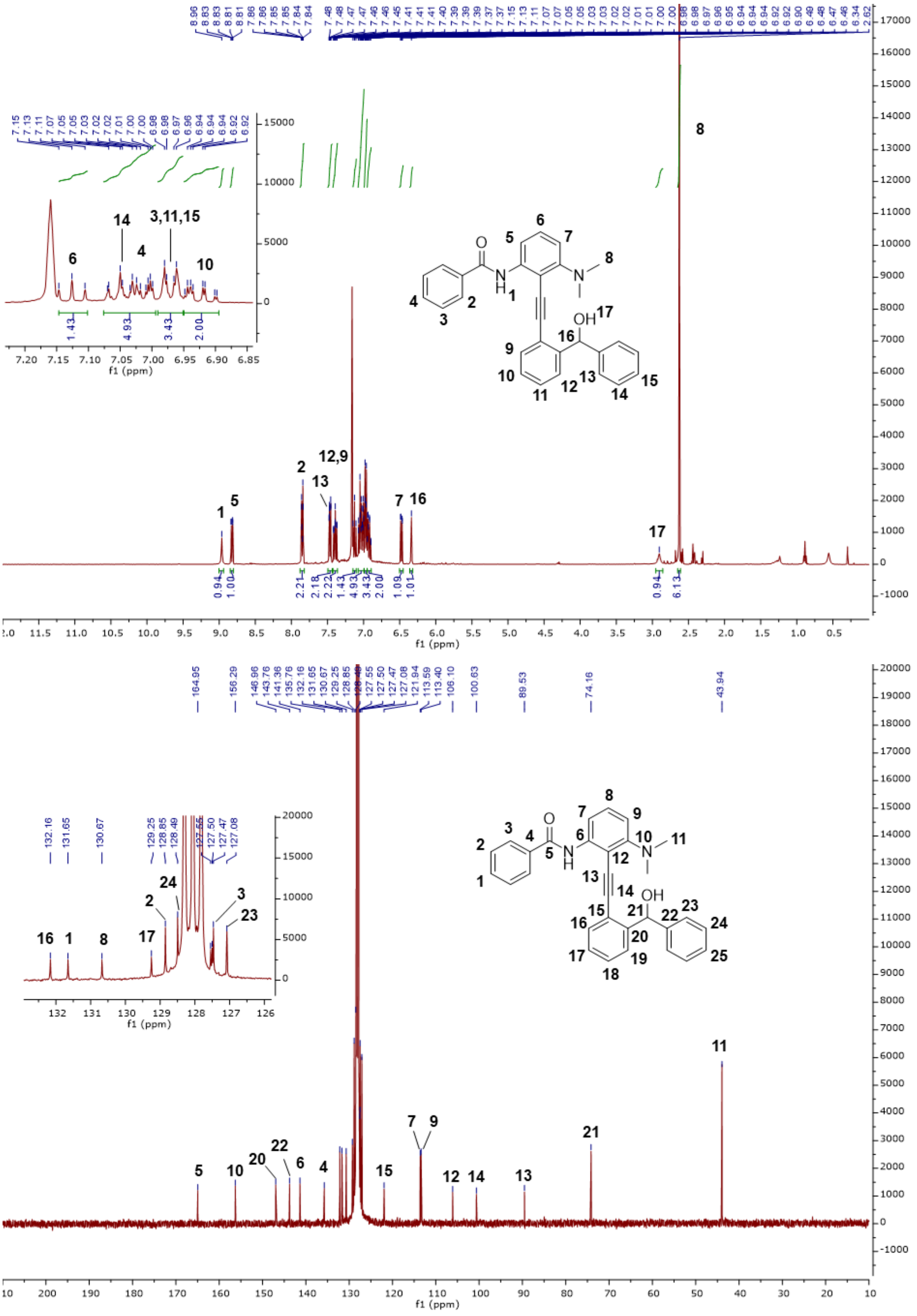

Figure S30: ^{1}H and ^{13}C NMR spectra of $\mathbf{2}_{\mathbf{alcohol}}$ at 0.045 M in C_6D_6.

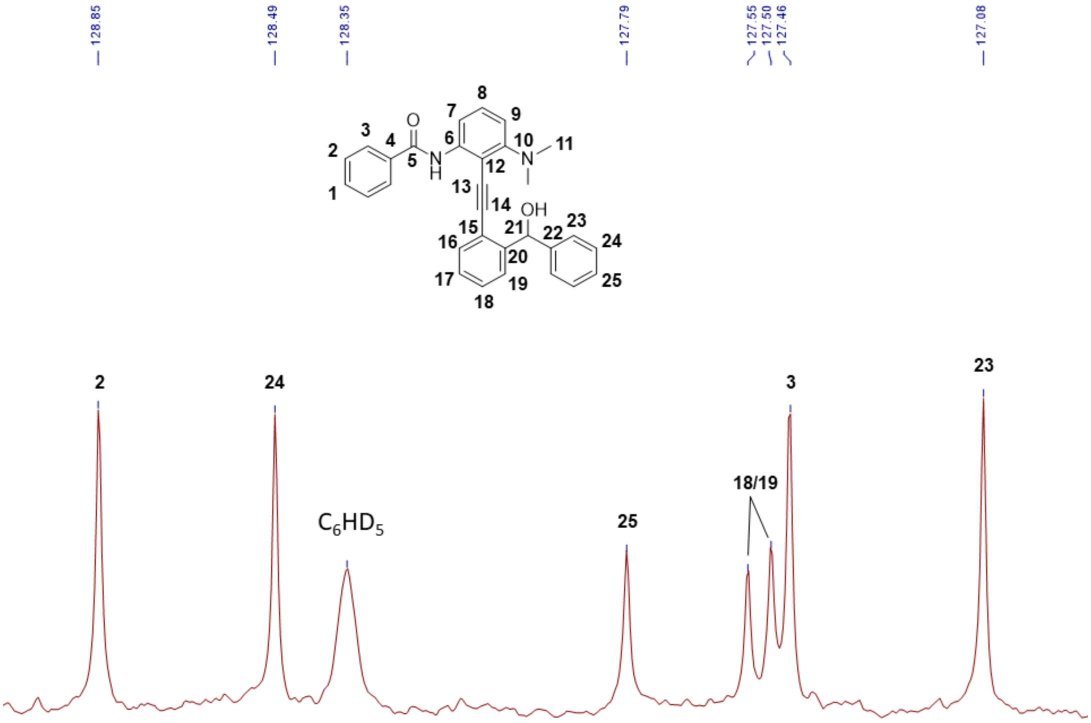

Figure S31: *DEPT 135 NMR spectra of* ***2****$_{alcohol}$ at 0.045 M in C_6D_6.*

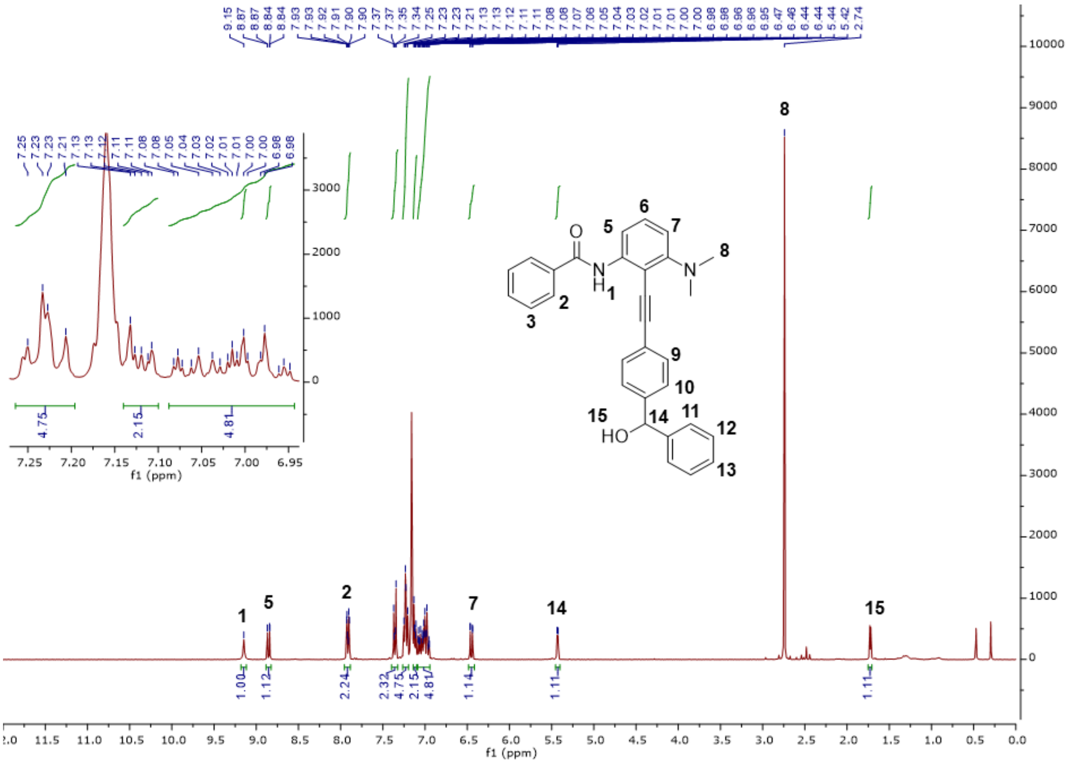

Figure S32: *^{1}H NMR spectra of* ***3****$_{alcohol}$ at 0.045 M in C_6D_6.*

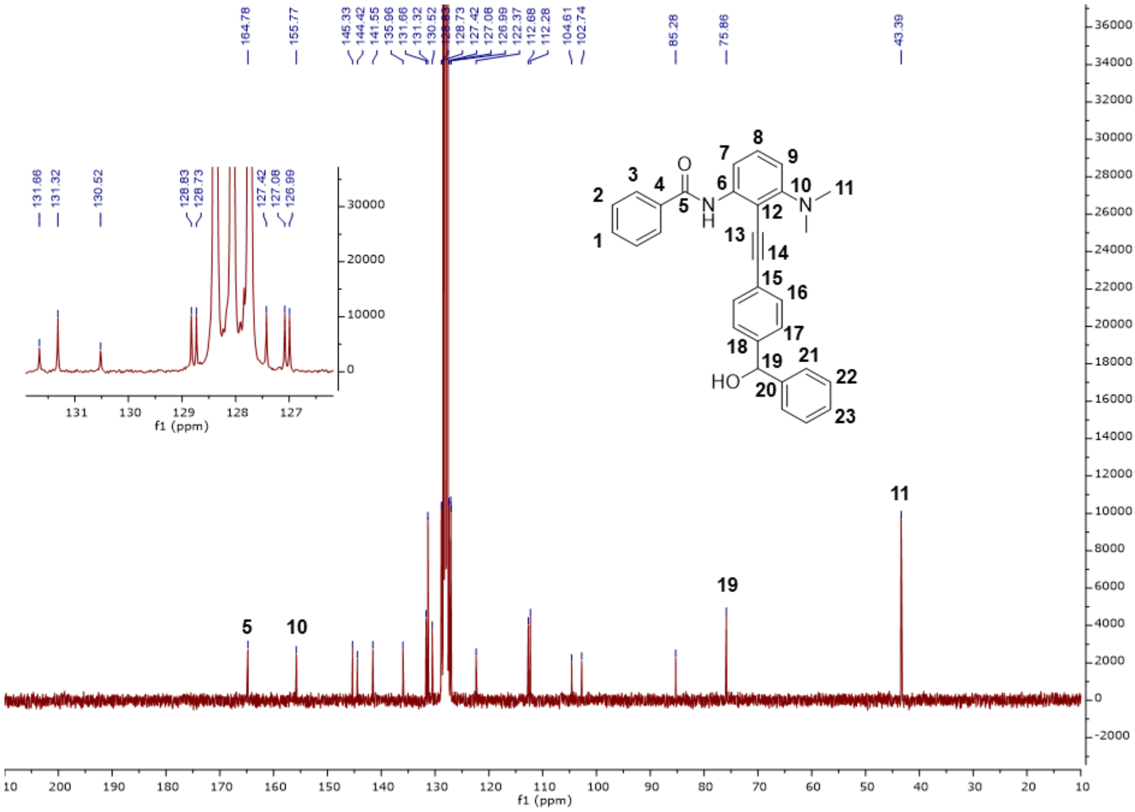

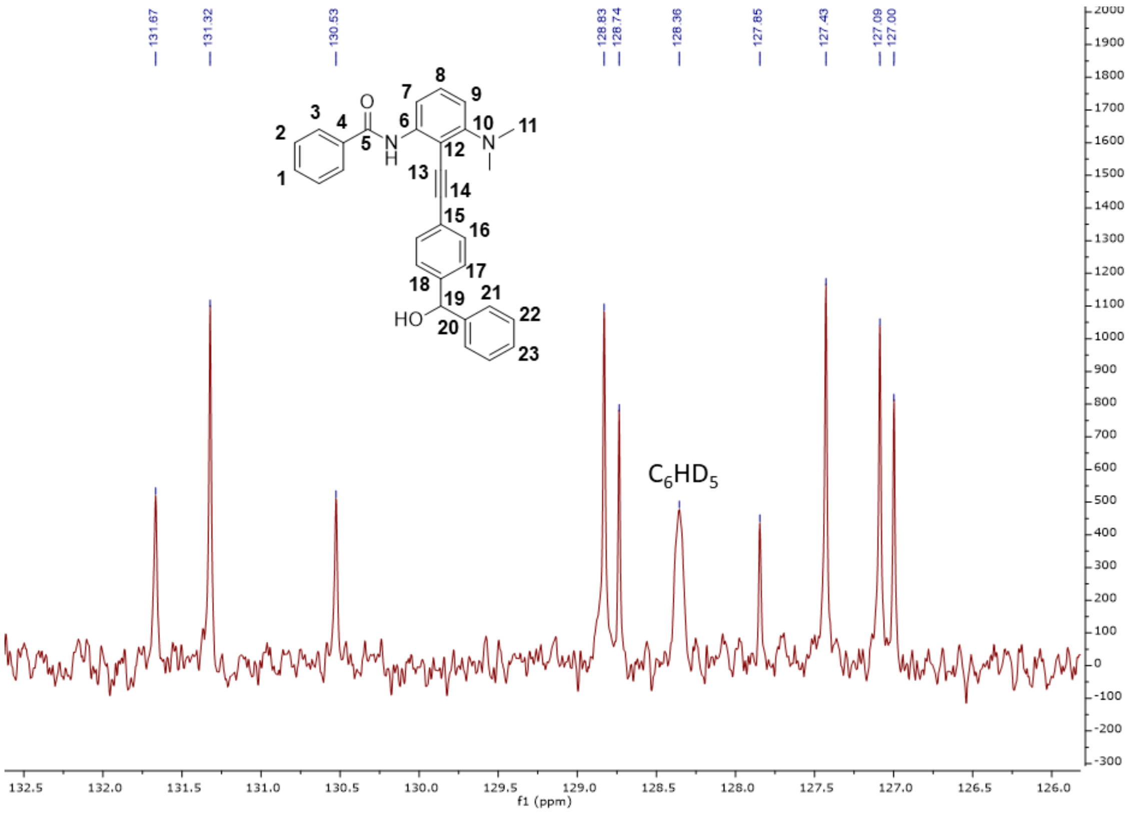

Figure S33: *^{13}C and DEPT 135 NMR spectra of **3**$_{alcohol}$ at 0.045 M in C_6D_6.*

References

1 R. Kawahara, K. Fujita and R. Yamaguchi, *J. Am. Chem. Soc.*, 2012, **134**, 3643–3646.

2 G. Evindar and R. A. Batey, *J. Org. Chem.*, 2006, **71**, 1802–1808.

3 M. Sienkowska, V. Benin and P. Kaszynski, *Tetrahedron*, 2000, **56**, 165–173.

4 I. M. Jones and A. D. Hamilton, *Org. Lett.*, 2010, **12**, 3651–3653.

5 P.-H. Li, L.-Z. Yu, X.-Y. Zhang and M. Shi, *Org. Lett.*, 2018, **20**, 4516–4520.

6 J.-M. L'Helgoual'ch, G. Bentabed-Ababsa, F. Chevallier, M. Yonehara, M. Uchiyama, A. Derdour and F. Mongin, *Chem. Commun.*, 2008, 5375.

7 W. Z. Yuan, H. Zhao, X. Y. Shen, F. Mahtab, J. W. Y. Lam, J. Z. Sun and B. Z. Tang, *Macromolecules*, 2009, **42**, 9400–9411.

8 J. Santhi and B. Baire, *ChemistrySelect*, 2017, **2**, 4338–4342.

9 N. Iwasawa, M. Shido, K. Maeyama and H. Kusama, *J. Am. Chem. Soc.*, 2000, **122**, 10226–10227.

10 S. Rigaut, J. Perruchon, S. Guesmi, C. Fave, D. Touchard and P. Dixneuf, *Eur. J. Inorg. Chem.*, 2005, **2005**, 447–460.

11 I. M. Jones, H. Lingard and A. D. Hamilton, *Angew. Chem. Int. Ed.*, 2011, **50**, 12569–12571.

12 AMPAC 11, 1992-2017 Semichem, Inc. 12456 W 62nd Terrace - Suite D, Shawnee, KS 66216.

13 Gaussian 16, Revision A.03, M. J. Frisch, G. W. Trucks, H. B. Schlegel, G. E. Scuseria, M. A. Robb, J. R. Cheeseman, G. Scalmani, V. Barone, G. A. Petersson, H. Nakatsuji, X. Li, M. Caricato, A. V. Marenich, J. Bloino, B. G. Janesko, R. Gomperts, B. Mennucci, H. P. Hratchian, J. V. Ortiz, A. F. Izmaylov, J. L. Sonnenberg, D. Williams-Young, F. Ding, F. Lipparini, F. Egidi, J. Goings, B. Peng, A. Petrone, T. Henderson, D. Ranasinghe, V. G. Zakrzewski, J. Gao, N. Rega, G. Zheng, W. Liang, M. Hada, M. Ehara, K. Toyota, R. Fukuda, J. Hasegawa, M. Ishida, T. Nakajima, Y. Honda, O. Kitao, H. Nakai, T. Vreven, K. Throssell, J. A. Montgomery, Jr., J. E. Peralta, F. Ogliaro, M. J. Bearpark, J. J. Heyd, E. N. Brothers, K. N. Kudin, V. N. Staroverov, T. A. Keith, R. Kobayashi, J. Normand, K. Raghavachari, A. P. Rendell, J. C. Burant, S. S. Iyengar, J. Tomasi, M. Cossi, J. M. Millam, M. Klene, C. Adamo, R. Cammi, J. W. Ochterski, R. L. Martin, K. Morokuma, O. Farkas, J. B. Foresman, and D. J. Fox, Gaussian, Inc., Wallingford CT, 2016.
